# ROS triggered local delivery of stealth exosomes to tumors for enhanced chemo/photodynamic therapy

**DOI:** 10.1186/s12951-022-01591-7

**Published:** 2022-08-23

**Authors:** Zhuo Wan, Xueqi Gan, Ruiyan Mei, Jianbin Du, Wen Fan, Mengying Wei, Guodong Yang, Weiwei Qin, Zhuoli Zhu, Li Liu

**Affiliations:** 1 Department of Hematology, Tangdu Hospital, Fourth Military Medical University, Xi’an, 710032 China; 2 State Key Laboratory of Oral Diseases, National Clinical Research Center for Oral Diseases, West China Hospital of Stomatology, Sichuan University, Sichuan 610041 Chengdu, China; 3 Department of Hepatobiliary Surgery, Xijing Hospital, Fourth Military Medical University, Xi’an, 710032 China; 4 State Key Laboratory of Cancer Biology, Department of Biochemistry and Molecular Biology, Fourth Military Medical University, Xi’an, 710032 China; 5 Shaanxi Department of National Clinical Research Center for Hematological Diseases, Xi’an, 710032 China; 6 Clinical Medical Research Center for Hematological Diseases of Shaanxi Province, Xi’an, 710032 China

**Keywords:** Exosomes, Cancer, Targeted delivery, ROS-responsive, Photodynamic therapy

## Abstract

**Background:**

Exosomes are recognized as effective platforms for targeted delivery for their high physicochemical stability and biocompatibility. However, most of the exosomes are inevitably and rapidly cleared by mononuclear phagocytic system (MPS) during cancer therapy. How to engineer exosome to enhance the delivery efficiency is being intensively explored. In this study, we have constructed mPEG2000-TK-CP05 decorated exosomes as effective delivery platforms to achieve enhanced photodynamic/chemical cancer therapy.

**Results:**

Exosomes were coated with CP05-TK-mPEG2000, in which CP05 is a peptide with high affinity to exosomal CD63 and TK could be cleaved by ROS. The resulted exosomes, namely stealth Exo, were electroporated to load RB (photosensitizer Rose Bengal) and Dox (Doxorubicin). We verified that the Stealth Exo@RB (Stealth Exo additionally loaded with RB) could escape MPS while accumulate in the tumor region efficiently in the xenograft model when laser irradiation conducted locally. Additionally, we revealed that the Stealth Exo serves as an efficient platform for Dox delivery. Dox, together with the RB mediated photodynamic therapy induce tumor cell damage synergistically in the tumor region. Moreover, the proposed switchable stealth exosomes minimized the dose of toxic Dox and thus allowed robust tumor immune response.

**Conclusions:**

Our results indicated that the proposed Stealth Exo greatly improves both the accessibility and efficiency of drug delivery, with minimal chemical or genetic engineering. The proposed Stealth Exo serve as a promising and powerful drug delivery nanoplatform in cancer treatment.

**Supplementary Information:**

The online version contains supplementary material available at 10.1186/s12951-022-01591-7.

## Background

Chemotherapy is one of the most classic methods of cancer treatment [1]. However, insufficient local drug concentration leads to poor chemotherapy effect or even drug resistance [2]. Increasing the dose of chemotherapeutic drugs will result in severe functional damage to important organs, thus seriously limiting the application of chemotherapy [3]. Doxorubicin, which is a highly effective anthracycline antibiotic for newly diagnosed DLBCL, has obvious and specific cardiotoxicity associated with prolonged exposure [4]. Therefore, local anti-tumor therapies aimed at increasing drug concentrations at tumor sites while avoiding systemic exposure are becoming increasingly attractive. Most research encapsulate doxorubicin in drug delivery vehicles such as exosomes to reduce the concentration of free drugs in the circulation [5–7]. However, nearly none drug delivery carrier could avoid unnecessary uptake by MPS.

Exosomes are nano-scale phospholipid bimolecular vesicles secreted by parental cells, can enter the blood stream and cross natural tissue barriers such as blood–brain barrier and have exactly effects for about four days after administration [8, 9]. More importantly, exosomes have a unique advantage of enhanced permeability and retention (EPR) effect [10]. Owing to these features, exosomes currently act as a novel and safe drug carrier to efficiently deliver various types of drugs such as siRNA, mRNA, bioactive drugs to tumors [11]. Emerging studies have applied exosomes to encapsulate chemical drugs such as paclitaxel and doxorubicin to treat cancers, which exhibited target cytotoxic effect on tumor cells while efficiently decreasing the adverse effects in other tissues [12, 13]. However, even if researchers used many approaches to decorate surface of exosomes to improve their specific tissue targeting, a great number of exosomes still can be endocytosed by mononuclear phagocyte systems, which results in a shorter blood circulation time and impaired cytotoxic effect on tumor cells [14, 15]. Modification of nano-particles (NP)’ surfaces with polyethylene glycol (PEG) has become a feasible method to extend the retention times and reduce the uptake by macrophages by preventing formation of a protein corona on exosomes [16, 17]. However, this approach will significantly decrease the uptake by tumor cells simultaneously. As a result, exploring a strategy to achieve PEGylation and selective de-PEGylation of exosomes is essential for efficient delivery. Herein, we have constructed a novel orientated exosome blasting system to overcome this dilemma. CP05 is a special peptide which can direct bind to the second transmembrane loop of CD63 with different moieties in exosomes irrespective of the origin of exosomes [18]. We have conjugated CP05 and PEG2000 via thioketal (TK) that is a ROS sensitive linker and will be degraded by ROS species. With CP05-TK-mPEG2000, exosomes can be surface functionalized with PEG via CD63-CP05 interaction, protecting exosomes from endocytosis by MPS and thus prolong the circulation time. Compared with normal tissues, ROS levels are often elevated in malignant tumors, which can partly cleave the TK linkers, divest PEG2000 and recover the surface of exosomes [19]. However, a challenging problem which arises in this field is that TK linkers can’t be degraded completely by physical levels of ROS.

Photodynamic therapy (PDT) has been applied to treat cancers for its little invasiveness, tiny toxicity in normal tissues and fewer side effect [20, 21]. With particular wavelength light, photosensitizers (PS) can be activated and generate active forms of oxygen as singlet oxygen (^1^O_2_), resulting in tumor cell death and inflammatory reactions [22]. Rose Bengal (RB) is a typical anionic photosensitizer which has high singlet oxygen quantum yield with 532 nm light irradiation [23]. Different from other types of photosensitizers that transfer hydrogen atoms to generate free radicals, RB can transfer its energy directly to oxygen once activated, leading to a more killing effectiveness [24].

In this study, we proved that exosomes decorated with PEG2000-TK-CP05 could efficiently escape the MPS and prolong the circulation in the blood. Moreover, we loaded RB into Stealth Exo to achieve efficient cleavage of thioketal under the irradiation of 532 nm laser. We have demonstrated enhanced tumor delivery efficiency of this stealth exosomes in DLBCL animal models when the coat was removed by photo-triggered ROS. This study has established a new strategy for exosome engineering towards precise and enhanced chemo‐photodynamic tumor therapy.

## Results

### Preparation and characterizations of stealth exosomes

**Fig. 1:**
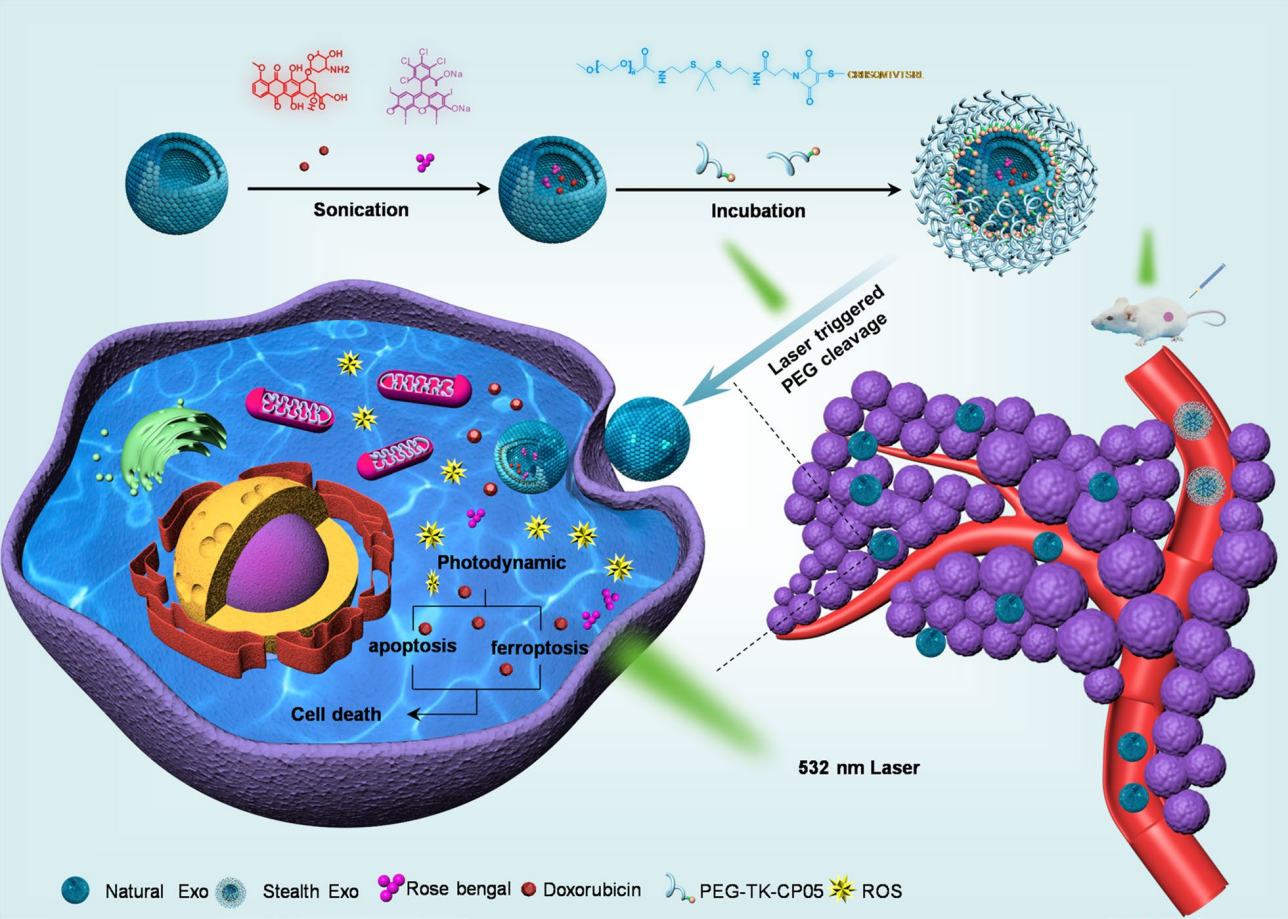
Schematic illustration of the study. Exosomes were first loaded with doxorubicin and Rose Bengal (RB) via sonication, followed by surface decoration with PEG2000-TK-CP05 for stealth. Under 532 nm laser local irradiation on the tumor region, RB-generated ROS cleave the TK and induce the burst release of exosomes. The local delivery of Doxorubicin (Dox), resulting in enhanced cancer therapy via multiple mechanisms

**Fig. 2:**
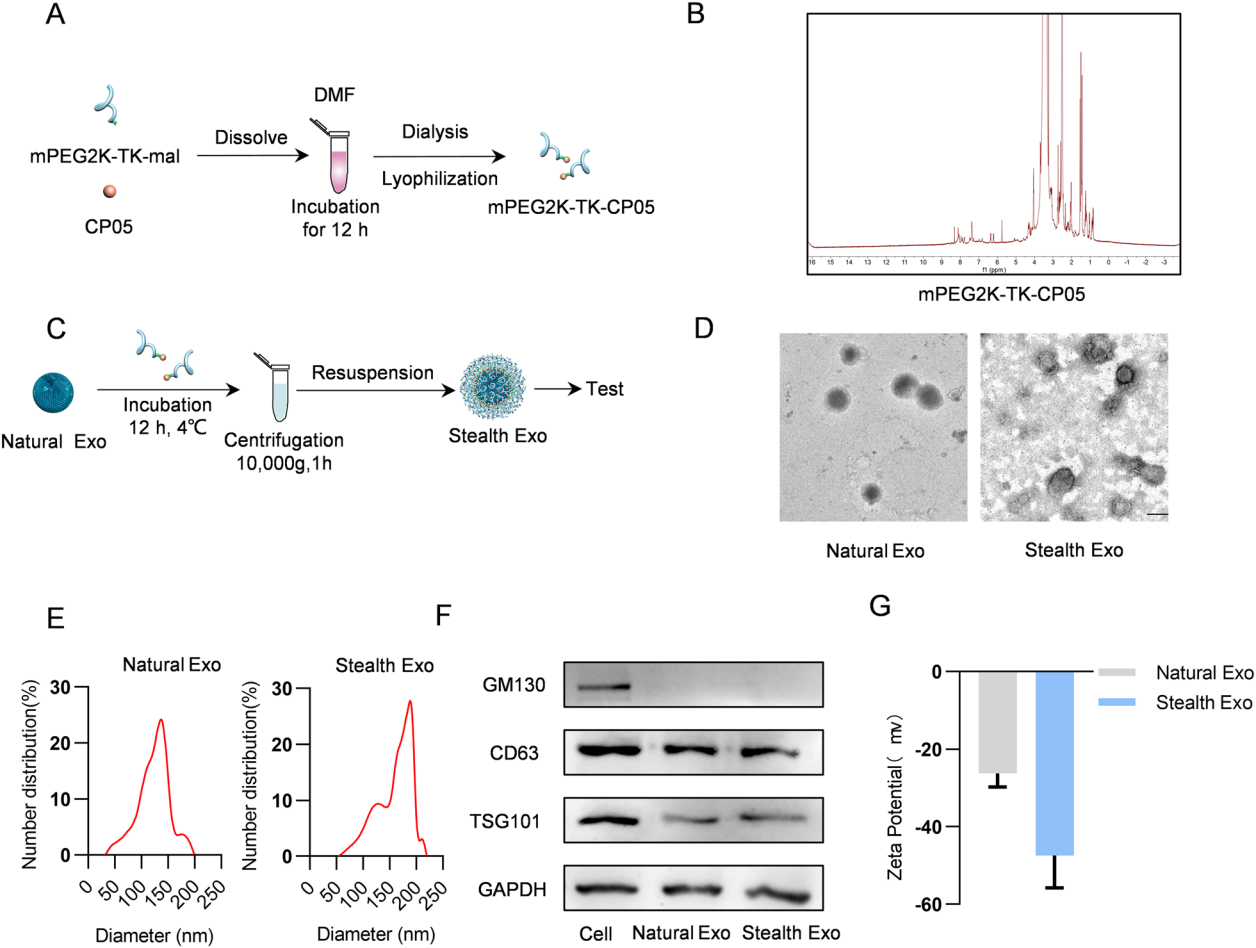
Construction and characterization of Stealth Exo. **A** Synthesis process for mPEG2K-TK-CP05. **B** ^1^H NMR spectra ((DMSO as solvent) of mPEG2K-TK-CP05. **C** Schematic diagram of the experimental procedure of Stealth Exo engineering. **D** Representative images of TEM analysis of Natural Exo and Stealth Exo. Scale bar = 100 nm. **E** Particle size distribution of Natural Exo and Stealth Exo as measured by DLS. **F** Western blot analysis of specific exosome marker proteins CD63, TSG101, CD81 and exclusive exosomal marker GM130 on donor cells, derived Natural Exo and Stealth Exo. GAPDH served as internal control. **G** Zeta potentials of Natural Exo and Stealth Exo

Exosomes have been reported to be readily taken up by a variety of cell types, especially by MPS, and most targeting specificity seems to be only subtle or unpredictable [25]. Studies have shown that PEG can strongly reduce the exosome-cell interaction via shielding the surface and protecting the exosome from aggregation, opsonization, and phagocytosis, thereby prolonging the retention in circulation [26]. In order to avoid exosome uptake by non-targeted cells and minimize the off-target effects to promote therapeutic efficacy, we herein designed a novel exosome delivery platform (Fig. 1). We firstly have synthesized mPEG2000-TK-MAL and CP05 peptide and the nuclear magnetic resonance (NMR) imaging showed the characteristic peaks respectively (Additional file 1: Fig. S1A, B). Then we conjugated these two substances via addition reaction (Fig. 2A) and the NMR results showed that mPEG2000-TK-MAL and CP05 peptide have been successfully connected (Fig. 2B). In this work, we used HEK293T cell as our donor cell for producing exosomes. We incubated exosomes and mPEG2000-TK-CP05 for 12 h at 4 °C, and then this mixture was centrifuged to remove the unbound mPEG2000-TK-CP05 to obtain the stealth exosome (Fig. 2C). As shown in Fig. 2D, both natural exosomes and stealth exosomes displayed the typical exosome morphology as revealed by transmission electron microscopy (TEM). NanoSight analysis revealed that the diameters of these exosomes ranged from 30 to 200 nm (Fig. 2E). The Western blot results showed that after modification, the expression level of GAPDH and other exosomal specific proteins were equal to the natural exosomes (Fig. 2F). It suggested that our modification process would not alter and damage the original characteristic of exosomes. In order to evaluate stability and integrity of stealth exosomes, zeta potential was recorded using PALS measurements. In our study, we found that stealth exosome had a higher negative surface charge (− 47.43 ± 8.38 mV) of the zeta potential compared with natural exosomes (− 26.25 ± 3.53 mV), which meant that the PEGylation of exosome would increase the dispersion stability (Fig. 2G). Such strong electronegativity can protect exosomes from endocytosis by MPS.

### Stealth exosome can escape internalization by MPS effectively

**Fig. 3:**
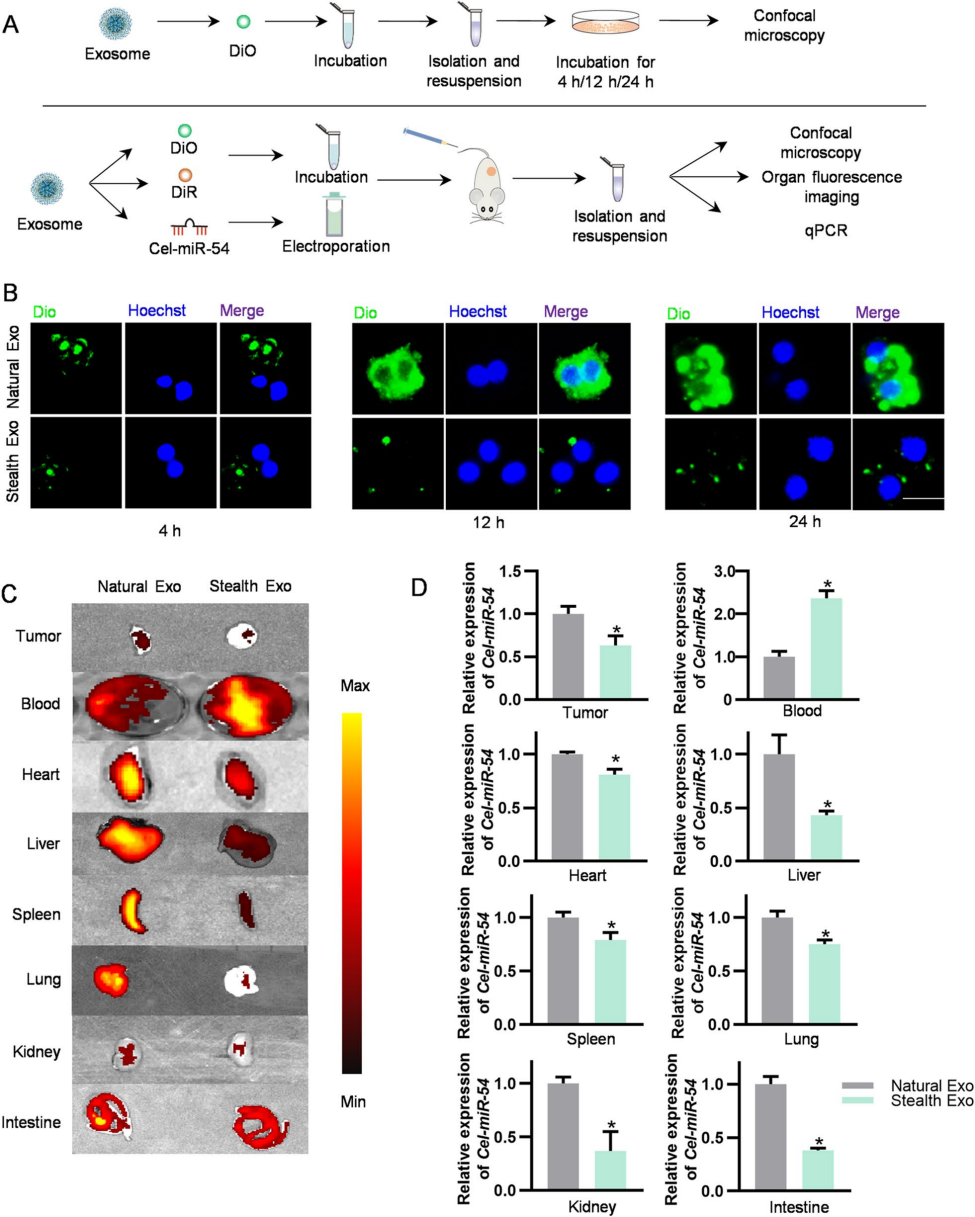
Efficient escape of Stealth exosome from the MPS. **A** Schematic illustration of these experiments. **B** Representative images show exosomes uptake by RAW264.7 cells. Different groups of exosomes were labelled with DiO and then incubated for 4 h/12 h/24 h. Cells were stained with Hoechst. Images were taken under fluorescence microscope. Scale bar = 20 μm. **C** Representative in vitro fluorescent images of the DiR-labeled exosomes in tumors and main organs. n = 6 mice. **D** qPCR analysis of relative expression of *Cel-miR-54* in various organs and tumors of mice treated with Natural Exo and Stealth Exo. *U6* served as an internal control. Data are expressed as mean ± SEM of six independent biological samples. *p < 0.05 by t-test

In order to test whether the constructed stealth exosome can escape the recognition by MPS, we have designed both in vitro and in vivo experiments (Fig. 3A). In vitro, we have incubated DiO -labeled Natural Exo and Stealth Exo with RAW 264.7 cells for 4/12/24 h respectively. As expected, in vitro fluorescence imaging showed that less amount of Stealth Exo phagocytosed by RAW 264.7 cells compared with the Natural Exo at all indicated time points (Fig. 3B). For in vivo test, we labeled exosomes with DiR and injected different type of exosomes into mice via the tail vein. After 4 h, 12 h, 24 h of administration, we have measured ex vivo fluorescent signal in different organs. Consistent with the in vitro test, ex vivo fluorescent images showed that most organs uptake less Stealth Exo compared with Natural Exo despite time lapse (Additional file 1: Fig. S2A), Additional file 1: Fig. S2B showed the quantitative data of ex vivo fluorescent images. Also, DiR-labeled exosomes were injected into the tumor bearing mice via the tail vein. The ex vivo fluorescent images showed that tumor tissues and other organs took less Stealth Exo, while Stealth Exo had more retention in blood compared with Natural Exo (Fig. 3C). Consistently, the confocal fluorescence images also exhibited that the uptake of Stealth Exo by different organs and tumors were much less than the Natural Exo (Additional file 1: Fig. S3). Cel-miR-54 is a miRNA from Caenorhabditis elegans, which had no homologue in mouse and human. Thus, cel-miR-54 is wildly used as an external reference spike-in miRNA [27]. Exosomes were loaded with *Cel-miR-54* mimics by electroporation, followed by tail vein injection to tumor bearing mice. As expected, the expression of *Cel-miR-54-5p i*n organs of Stealth Exo group was significantly less than that in Natural Exo group. On the contrary, the expression of *Cel-miR-54-5p* in serum of Stealth Exo group was much higher than that in Natural Exo group (Fig. 3D). These data were in coincidence with our assumptions that exosomes decorated with PEG could escape the recognition of MPS and have a longer circulation time.

### Tumor targeting of Stealth Exo@RB under the irradiation of 532 nm laser

**Fig. 4:**
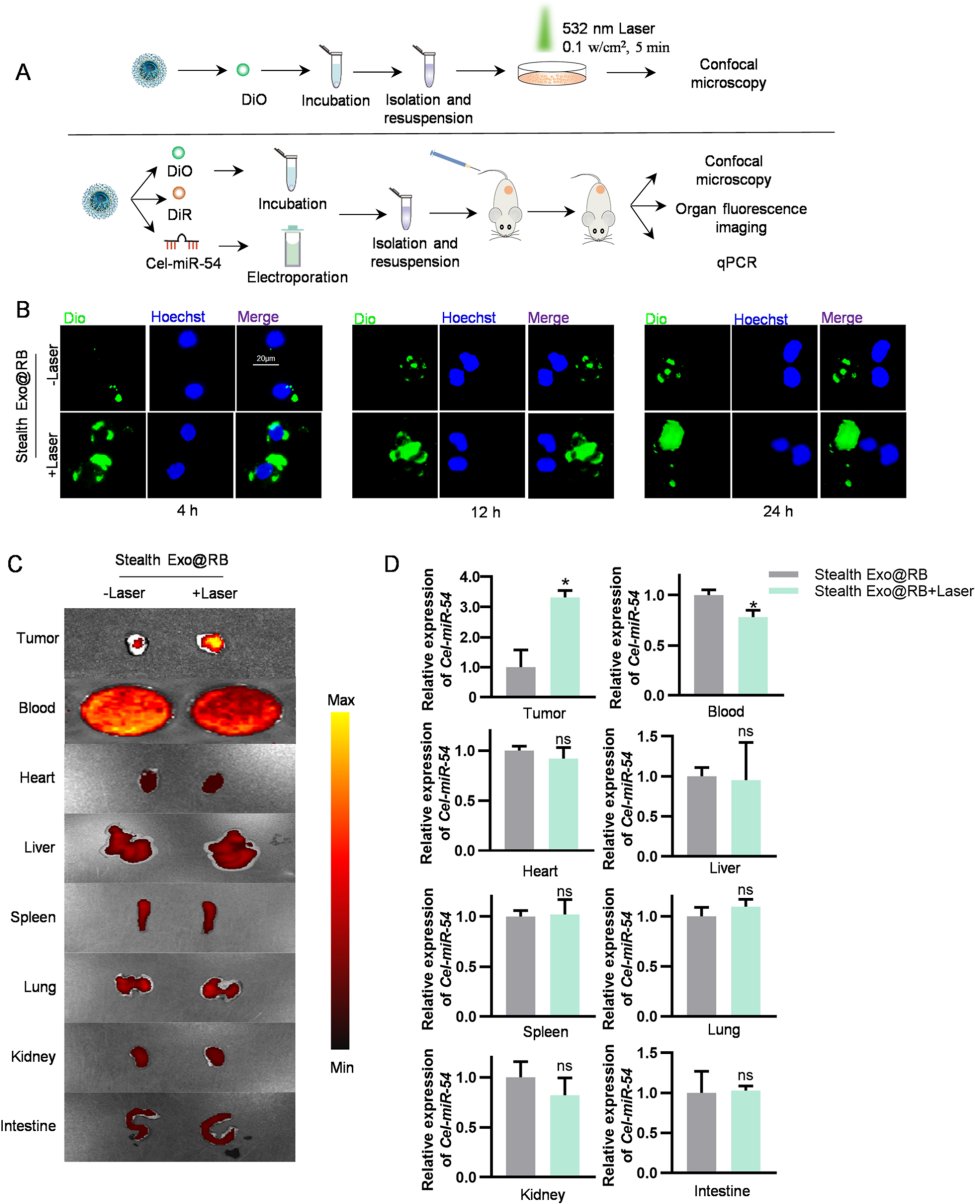
Tumor targeting of Stealth Exo@RB under the irradiation of 532 nm laser. **A** Schematic illustration of these experiments. **B** Representative images show exosomes uptake by Raw264.7 cells. Different groups of exosomes were labelled with DiO and then irradiated by 0.1 w cm^−2^ 532 nm laser for 5 min. After 4 h/12 h/24 h, Images were taken under fluorescence microscope. Scale bar = 20 μm. **C** Representative in vitro fluorescent images of the DiR-labeled exosomes in tumors and main organs. n = 6 mice. **D** qPCR analysis of relative expression of *Cel-miR-54* in various organs and tumors in mice treated with Stealth Exo and Stealth Exo + Laser. U6 served as an internal control. Data are expressed as mean ± SEM of six independent biological samples. *p < 0.05 by t-test

As shown in Fig. 3C and Additional file 1: Fig. S3, we didn’t observe a detectable increase in tumor accumulation, which was consistent with the previous studies. In order to remove the PEG decoration locally to achieve tumor targeting, we have linked a TK bond between mPEG2000 and CP05, which can result in de-PEGylation of Stealth Exo via ROS-mediated cleavage of TK (thioketal bond). RB is a typical anionic photosensitizer which has high singlet oxygen quantum yield with 532 nm light irradiation. In order to test the encapsulation rate, we loaded RB into Natural Exo via sonication to acquire Natural Exo@RB and then centrifuged the mixture (Additional file 1: Fig. S4A). By detecting the absorbance in the supernatant, we indirectly determined the loading efficiency. Additional file 1: Figure S4D showed the maximum encapsulation rates of RB could reach 82.09 ± 5.21%. The maximum absorption peak of RB in PBS is about 540 nm (Additional file 1: Fig. S4B). Additional file 1: Figure S4C showed that the intensity and concentration of the maximum absorption peak were linearly related in a certain concentration range. To obtain Stealth Exo@RB, we first loaded RB into Natural Exo to obtain Natural Exo@RB, then we incubated Natural Exo@RB with mPEG2000-TK-CP05. The character of Stealth Exo@RB was shown in Additional file 1: Fig. S5A, B. Theoretically, encapsulation of Cel-miR-54 will interfere with the encapsulation rate of RB because they are both water soluble. However, this procedure was only for exosome tracing in this study, which would have no effect on functional test. To provide further insight into Stealth Exo@RB based targeted therapeutic delivery, we tested the targeting ability of DiO/DiR labeled Stealth Exo@RB and *Cel-miR-54* encapsulated Stealth Exo@RB under the 532 nm laser irradiation (Fig. 4A). In vitro, we observed that Raw264.7 cells could take in much more DiO-labeled Stealth Exo@RB when irradiated with 0.1 w cm^−2^ laser for 5 min (Fig. 4B). Confocal images showed that prolonging the incubation time would not increase the additional uptake of exosomes, which indicated that the cell uptake of exosomes has reached a saturated state (Fig. 4B). In vivo, DiO/DiR-labeled exosomes and *Cel-miR-54* encapsulated exosomes were injected into the tumor bearing mice via the tail vein respectively. In the laser group, the tumors were irradiated by 0.5 w cm^−2^ 532 nm laser for 15 min at 4 h post-injection. Mice were sacrificed after irradiation for further detection. Ex vivo fluorescent images displayed that laser irradiation on tumor site would only increase the Stealth Exo@RB accumulation locally compared with no laser group. And the fluorescent signal in serum decreased in the laser group (Fig. 4C), semi-quantification of fluorescence intensity of different organs was presented in Additional file 1: Fig. S6B. The qPCR test and the confocal images of tumor and organs slices showed the same results (Fig. 4D and Additional file 1: Fig. S6A). These results verified Stealth Exo@RB combined with PDT as a potential ideal nanocarrier delivery strategy for prolonging circulation, decreasing nonspecific uptake by the MPS and simultaneously targeting tumor with high efficiency.

### Synergistic photodynamic chemotherapy effects of Stealth EXO@RB/Dox in vitro

**Fig. 5:**
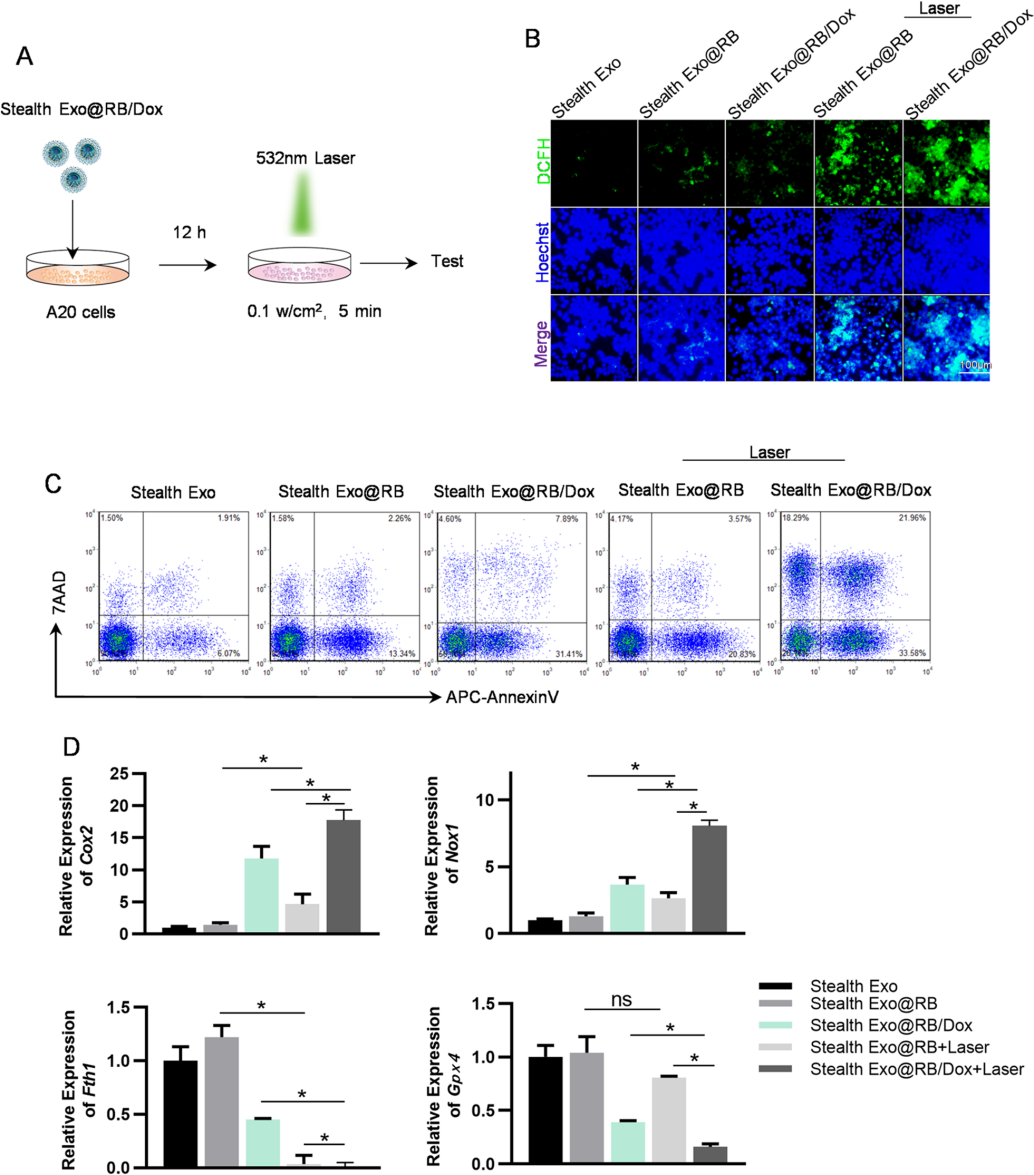
Efficient cell death induction by Stealth Exo@RB/Dox upon laser irrdatiation in vitro. **A** Schematic illustration of the procedure. A20 cells were treated with indicated exosomes and laser irradiation (532 nm laser, 0.1 W cm^−2^, 5 min). **B** Fluorescence images of ROS level analyzed by DCFH-DA staining in A20 cells with indicated treatments. Scale bar = 100 μm. **C** FCM analysis of cell death by Annexin V-APC/7-AAD double staining in cells treated same as above. **D** qPCR analysis of ferroptosis related genes in A20 cells treated as above. *Gapdh* served as internal controls. Data are expressed as mean ± SEM of three different experiments. *p < 0.05 by one-way ANOVA

The efficient in vivo tumor targeting effect of the Stealth EXO@RB combined PDT encouraged us to investigate its therapy potential on tumor xenograft mice when carried with chemotherapy drugs. Dox is a highly effective anthracycline antibiotic for DLBCL. In order to obtain Stealth Exo@ Dox and test the encapsulation rate, we loaded Dox into Natural Exo via sonication to acquire Natural Exo@Dox (Additional file 1: Fig. S7A). By detecting the absorbance in the supernatant, we indirectly determined the loading efficiency of drug by exosomes. The maximum absorption peaks of Dox in PBS are 480 nm (Additional file 1: Fig. S7B) and the intensity and concentration of the maximum absorption peak were linearly related (Additional file 1: Fig. S7C). Additional file 1: Figure S7D showed the maximum encapsulation rates of Dox could reach 75.6 ± 6.77%. Next, RB were loaded into Natural Exo@Dox as previously mentioned. Then, Natural Exo@RB/Dox were incubated with mPEG2000-TK-CP05 to obtain the Natural Exo@Dox. RB is hydrophilic and loaded inside of the exosome, while DOX is hydrophobic and loaded on the surface of the exosome. Therefore, simultaneously loading RB and DOX into exosomes will not affect each other. We investigated the synergistic photodynamic chemotherapy effects of Stealth EXO@RB/Dox combined irradiation. In vitro, we incubated exosomes (2 mg mL^−1^, 50 µL) with A20 cells for 12 h, then we irradiated cells with 0.1 w cm^−2^ 532 nm laser for 5 min, the whole process was shown in the Fig. 5A. The ROS level was measured by DCFH-DA kit. The level of ROS in A20 cells treated with Stealth Exo@RB/Dox combined laser was significantly higher than all other groups. Additionally, laser irradiation would significantly increase the ROS level in Stealth Exo@RB group (Fig. 5B). In contrast, exosomes alone or laser irradiation alone had no obvious effect on ROS level of A20 cells (Fig. 5B, Additional file 1: Fig. S8A). Cell death was analyzed by Annexin V-APC/7AAD dual staining assay. As shown in Fig. 5C, the percentages of total necrotic and apoptotic cells were 9.48%, 17.18%, 43.90%, 28.57%, 73.83% in Stealth Exo, Stealth Exo@RB, Stealth Exo@RB/Dox, Stealth Exo@RB (L) and Stealth Exo@RB/Dox (L) groups respectively. There were about 20% Annexin V^−^/7AAD^+^ cells in Exo@RB/Dox with laser irradiation group, which should be explained by the fact that RB/Dox/Laser treatment (resulted in enhanced Dox mediated chemotherapy compared with other groups) not only induces apoptosis, but also causes other types of cell death, including necrosis and ferroptosis. CCK8 assay also proved that cells in Stealth Exo@RB/Dox (L) groups have the lowest viability, which was consistent with the finding of flow cytometry (Additional file 1: Fig. S11D). Notably, laser irradiation alone had no effect on cell death (Additional file 1: Fig. S8B). Dox not only can directly interact with topoisomerase II-β and induce double strand DNA breakage, but also can induce the disturbance of calcium homeostasis, mitochondrial iron accumulation, and excess production of ROS. Dox can induce both apoptosis and ferroptosis [28, 29]. By qPCR test, we found that ferroptosis related genes such as *Nox1* and *Cox2* were overexpressed significantly in the group of Stealth Exo@RB/Dox, Stealth Exo@RB (L) and Stealth Exo@RB/Dox (L), while the expression of other ferroptosis related genes such as *Gpx4* and *Fth1* were decreased. Consistent with the flow cytometry results, cells in Stealth Exo@RB/Dox (L) group exhibited the most severe ferroptosis (Fig. 5D). Also, cells in the laser alone group have no significant change in ferroptosis related genes (Additional file 1: Fig. S8C). These results above suggested that Stealth EXO@RB/Dox combined laser treatment had an excellent synergistic photodynamic chemotherapy effect.

### Synergistic photodynamic chemotherapy effects of Stealth EXO@RB/Dox in vivo

**Fig. 6:**
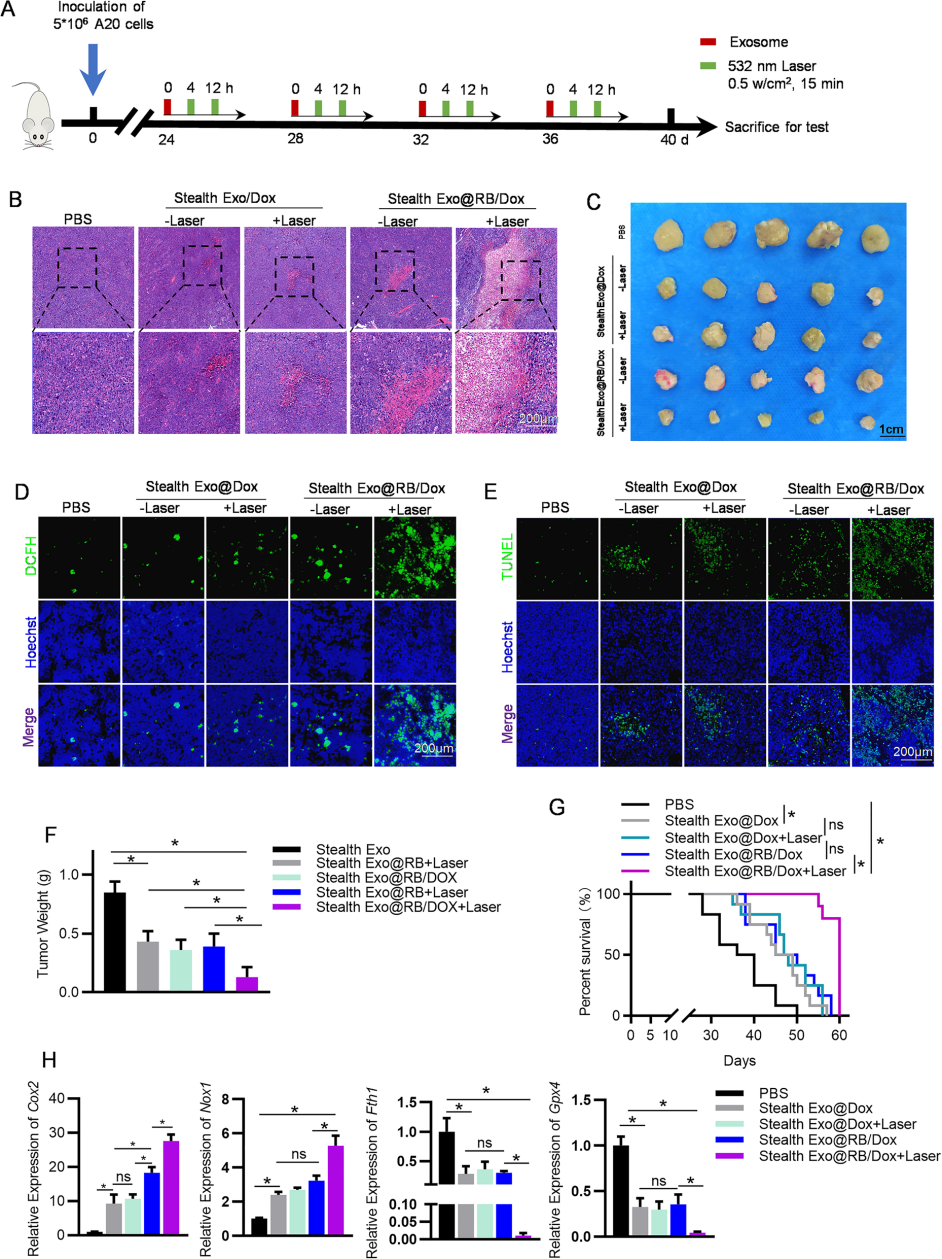
Laser irradiation induces robust therapeutic effects of Stealth Exo@Dox in vivo. A Schematic of experimental design. Balb/c mice were implanted with 5 × 10^6^ A20 cells subcutaneously. Exosomes were injected at the 24th day after tumor cell implantation. 532 nm laser irradiation (0.5 W cm^−2^, 10 min) was performed on the tumor region right after exosome injection and at the 4 h and 12 h post-injection. **B**–**D** Representative images of histological analysis of the tumor tissues in mice with indicated treatments. Mice were sacrificed after twice treatments. H&E (**B**), DCFH (**C**), and TUNEL (**D**) were performed. Scale bar = 200 μm. n = 6 for each group. **E** qPCR analysis of ferroptosis related genes in tumor tissues in different groups. *Gapdh* served as internal controls. Data shown are representative of 6 mice and expressed as mean ± SEM. *p < 0.05 by one-way ANOVA. **F** Representative images of excised tumors in mice treated as indicated. Mice were sacrificed at the end of the experiment. n = 10. **G** Tumor volume of tumors on Balb/c mice post various treatments (n = 10). **H** The survival curve of tumor-bearing Balb/c mice with different treatments. (n = 10 mice/group). *p < 0.05

In the following experiments, we explored the therapeutic effect of Stealth Exo@RB/Dox combined with laser irradiation in the A20 cells bearing mice models. To ensure exosomes entering into tumor cells efficiently, laser irradiation was performed to remove the PEGylation via photo-dynamics induced ROS. Exosomes can accumulate in the tumor tissues via the enhanced permeation and retention (EPR) effect with time. To this end, laser irradiation needs to be performed multiple times. Thus, animals received tail vein injections of PBS, Stealth Exo@Dox, Stealth Exo@RB/Dox (2 mg mL^−1^, 200 µL) every 4 days. In the laser group, tumors were treated with 532 nm laser (0.5 w cm^−2^) for 15 min at 4 h and 12 h post-injection. All the mice were sacrificed at the 40th day after tumor bearing (Fig. 6A). The images in Fig. 6C were typical tumors which were collected at the end of the treatment, providing direct insight into the antitumor efficacy of the different treatments. Also, Fig. 6F that presented tumor weights after different treatments showed that Stealth Exo@RB/Dox combined laser group have the strongest anti-tumor effect. Kaplan–Meier with log-rank test was conducted to assess the survival time of the experimented animals, of which the results were shown in Fig. 6G. Less than 50% mice survived longer than 36 d in the group of PBS, whereas the animal survival time in the Stealth Exo@RB/Dox combined laser group was prolonged and was improved compared with PBS group. Importantly, mice in Stealth Exo@RB/Dox combined laser group showed the best efficiency at tumor growth inhibition and showed the longest survival time. H&E staining images also showed that tumors in the Stealth Exo@RB/Dox combined laser group had more cell necrosis and inflammatory cell infiltration (Fig. 6B). Furthermore, in the tumor tissues, we evaluated apoptosis by using TUNEL and ROS level by DCFH-DA kit. DCFH staining showed that cells in Stealth Exo@RB/Dox combined laser group had the highest ROS level (Fig. 6D). In Fig. 6E, the highest cell apoptosis rate also occurred in the tumors treated with Stealth Exo@RB/Dox combined laser. Consistently, the changes in the expression of ferroptosis related genes in tumor issues further confirm the synergistic effect of photodynamic chemotherapy on tumor growth inhibition (Fig. 6H). Moreover, compared with Stealth Exo@Dox and Stealth Exo@RB/Dox alone group, C11 BODIPY 581/591 staining of tumor issues showed that Stealth Exo@RB/Dox combined laser would lead to the most severe lipid peroxidation of tumor cells, which would enhance Dox induced ferroptosis (Additional file 1: Fig. S9). In order to test whether this photodynamic chemotherapy could mediate the immune response in tumor site, we measured the mRNA expression level of *Il-2*, *Il-6*, *Il-10*, *Tnf-α*, *Tgf-β* and *Inf-γ* in tumor issues. As shown in Additional file 1: Fig. S10A-F, the expression of *Ifn-γ*, *Il-2*, *Tnf-α*, *Il-6* which encoded T cell activation cytokines were upregulated in tumors treated with Stealth Exo@RB/Dox and Laser, while the expression of *Tgf-β* and *Il-10* which were immunosuppressive genes were downregulated. Compared with no laser group, PBS group and Dox groups, laser irradiation on tumor issues would significantly increase the expression of anti-tumor immune genes. These results demonstrated that not only this strategy killed tumor cells by large quantities of ROS and chemotherapy, but also induced anti-tumor immune response.

**Fig. 7:**
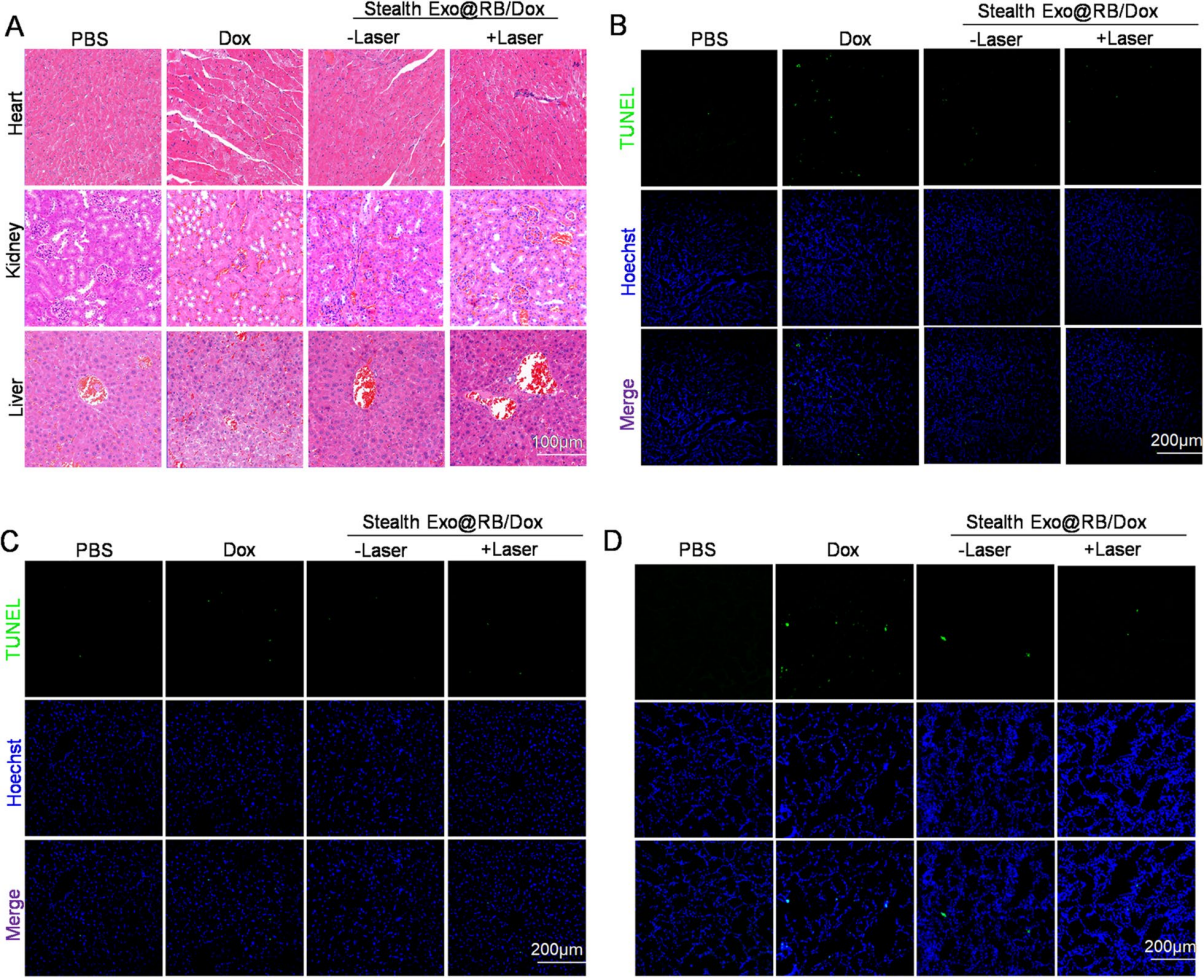
Side-effects of the Stealth Exo delivery system. **A** Representative images of the H&E staining of different organs from the A20-bearing mice with indicated treatments. Scale bar = 100 μm. **B** Representative images of the TUNEL staining of different organs from the A20-bearing mice with indicated treatments. Scale bar = 200 μm. n = 6 for each group

For the purpose of evaluating the biosafety of Stealth Exo@RB/Dox in vivo, PBS, Dox (4 mg/kg), Stealth Exo@RB/Dox (2 mg mL^−1^, 200 µL) were injected into the tail vein of BALB/c mice. The free concentration of Dox was the same as the Dox loaded into exosomes. The toxicity of Stealth Exo@RB/Dox to major organs was investigated by H&E staining and TUNEL staining. As shown in Fig. 7A–D, consistent with previous studies, Dox induced significant apoptosis in the heart, liver and kidney. Though we observed little apoptosis cells in Stealth Exo@RB/Dox group (both with laser and without laser) compared with PBS group, the organ damage was much less severe than that of the Dox group. These results above indicated that our proposed Stealth Exo@RB/Dox is a biocompatible nanocarrier without obvious side effects in vivo, which is much suitable for clinic.

## Discussion

In this study, we have successfully constructed a photodynamically induced orientated blasting system of stealth exosomes and verified the delivery and treatment advantages in tumor bearing mice. The decoration of exosomes via PEG2000-TK-CP05 could significantly reduce the exosome uptake by MPS and extend its blood circulation time. More importantly, these Stealth Exo@RB exhibit more exosome uptake and tumor distribution in mice under the radiation of 532 nm laser, which means this system not only can limit the passive phagocytosis of exosomes in the body by the MPS but also realize the active targeting function of exosomes at the tumor site simultaneously. Animal experiments also demonstrated that this delivery system could efficiently deliver doxorubicin and RB into tumor site at the excitation of laser, which simultaneously triggered apoptosis and ferroptosis of tumor cells. More promising, this platform showed tiny cytotoxicity and better biological safety.

During the past decade, great efforts have been made to the research of optimizing targeted delivery efficiency of exosomes and controlled drug release. Many works attribute to modifying exosome surface with specific homing ligands or peptides for different organs to improve the targeting ability. Tian et al. have decorated immature dendritic cells with iRGD peptide and applied these modified exosomes as drug carriers to target αV integrin-positive tumor cells [30]. Li et al. [31] also showed that macrophage-derived exosomes modified with a c-Met binding peptide and loaded with DOX-NPs exhibit significant immune evading ability and tumor-targeting capability. However, even if the distribution of modified exosomes in tumor site has increased, most of the exosomes will still be swallowed by MPS organs such as liver and spleen, resulting in unnecessary drug accumulation and organ damage. In addition, these medications might result in unnecessary molecule changes on the membrane of exosomes and induce immunogenicity. In our study, we coated exosomes with TK-mPEG2000, which not only endow exosomes with such “stealth” properties but also can reduce the immunogenicity and toxicity of exosomes. TK-mPEG2000 was anchored onto the exosome surface via CD63-CP05 interaction, which has many advantages as follows. Firstly, CD63 is the marker of exosomes and CP05–CD63 interaction ensures nearly all the exosomes being surface functionalized. Secondly, CP05-TK-mPEG2000 is easy for manipulation and the surface functionalization can be achieved by direct incubation. In addition, compared with DSPE-PEG and the analogues, which directly incorporated into exosomes, the CP05–CD63 interaction strategy has minimal effects on the exosome surface. From this perspective, decorating exosomes with PEG is more promising in clinic use compared with protein modification. Liu et al. [32] have constructed a platform for tumor chemo‐photodynamic therapy based on dual ligand functionalized, AuNR‐conjugated exosomes. Under NIR light, AuNRs attached to the exosomes could impact the permeability of exosomes membrane and enhance drug release. Similarly, we proved that RB pre-loaded in stealth exosomes could be activated by 532 nm laser and release heat and ROS. On the one hand, ROS cleaved TK linkers and removed the PEG modification on the surface of exosomes, allowing more penetration and uptake in tumor issues. One the other hand, high yield of ROS produced by RB could enhance apoptosis and ferroptosis triggered by doxorubicin, which is consistent with previous study [33–35]. These above suggest that this delivery system perfectly integrates the targeting strategy and the therapeutic strategy, which makes full use of the limited space in exosomes by loading RB. Traditional chemotherapy methods usually inevitably lead to the reduction of anti-tumor immune response caused by bone marrow suppression. Interestingly, in our study, we have observed an obviously increasing expression of anti-tumor immune-related genes in tumor issues after mice treated with Stealth Exo@RB/Dox combined with laser. T cell subset analysis such as CD4^+^ αβT cells and CD8^+^ αβT cells would be more convinced and we would like to conduct this experiment in the further study. Thus, we speculate that the chemo-photodynamic therapy induced tumor antigen released of dead tumor cells can trigger anti-tumor immune reaction, which may contribute partly to tumor therapy.

Even if our delivery strategy can effectively and accurately kill tumors with minimal side effects, we still have many aspects to improve and optimize. For instance, although 532 nm laser have the advantages of high power and short action time, which will not produce non-selective heating of the skin tissue around the target tissue, the penetration ability of green lasers is still inferior to long wavelength lasers. This factor limits the application of this delivery system to superficial lymphomas rather than lymphomas occur in deep tissues. Activating RB with a specific frequency of sonication or guiding the green light to deep tissue with a tiny optical fiber may be effective ways to solve this dilemma. In addition, clearing the contents of the original exosomes such as nucleic acids, proteins, etc. to expand the loading space in the exosomes may be significant to improving the encapsulation rate of drugs into exosomes, which will also avoid the passive influence of exosomal contents on the treatment effect.

## Conclusion

In summary, we here have constructed a switchable stealth exosome via photo-triggered local removal of PEGylation on the stealth exosomes. The system delivers the doxorubicin and Rose bengal destined to the tumor sites under the activation of 532 nm laser. It would be promisingly expected that the strategy can be applied in the treatment of multiple superficial tumors such as some kinds of lymphoma, breast cancer, and melanoma.

## Methods

### Cell culture

HEK293T cells and RAW 264.7 cells were purchased from Procell Biotechnology (China). A20 were donated by Dr. He from Department of Pharmacy of the Fourth military medical university. HEK293T, RAW 264.7 cells were cultured in complete media containing high glucose Dulbecco's modified Eagle medium (DMEM) with 10% fetal bovine serum and 1% penicillin/streptomycin (Hyclone) in a humidified incubator with 5% CO_2_ at 37 °C, A20 cells were cultured in complete media containing RPMI 1640 with 10% fetal bovine serum and 1% penicillin/streptomycin (Hyclone).

### Mice

BALB/c (6–8 weeks old) male mice were purchased from Animal Center of the Fourth Military Medical University. The animal experimental and housing procedures were performed following the guidelines approved by the Animal Experimentation and Ethics Committee of Fourth Military Medical University. A20 cells (5 × 10^6^ cells suspended in 200 μL PBS) were subcutaneously injected on the right back of mice for subcutaneous DLBCL model. The long diameter (L) and short diameter (S) of tumor were measured by digital vernier caliper. Tumor volume was assessed as (L × S^2^) × 0.5.

### Exosome isolation and characterization

For the isolation of exosomes from HEK293T cells, the cell culture medium was replaced with serum-free medium 48 h before collection. Then cell supernatants were collected and centrifuged at 250×*g* for 10 min to eliminate dead cells and then at 3000×*g* for 15 min to remove residual cellular debris. The resulting supernatants were then filtered through 0.22 μm filters to remove adhesives. Then exosome isolation was performed using ultracentrifuge method as described before [36]. The extracted exosomes were re-suspended in PBS and stored at − 80 °C before using.

The isolated exosomes were diluted to 1 μg mL^−1^ and the size distribution was analyzed by NanoPlus (Otsuka Electronics, Japan). The morphology of exosomes was analyzed by electron microscopy. The exosome suspension was added onto the copper mesh. Then the exosomes were then stained with 1% uranyl acetate for 1 min and imaged by the electron microscope (JEM-2000EX TEM, JEOL Ltd, Tokyo, Japan). Western blot was performed to identify the specific biomarkers of exosomes. Normal procedures were the same as previously described [37]. Primary antibodies used in the study included anti-GM130 (1:1000, Abcam, ab30637), anti-TSG101 (1:500, Santa, sc-7964), anti-CD81 (1:1,000, Abcam, ab109201), anti-CD63 (1:1,000, Abcam, ab134045) and anti-GAPDH (Proteintech, 60004-1-1g). Second antibodies used in the study were HRP-conjugated goat anti-rabbit IgG (1:5000, Cell Signaling Technology, 7074P2) and HRP-conjugated goat anti-mouse IgG (D110087, BBI, China).

### Synthesis of mPEG2000-TK-CP05

mPEG2000-TK-mal and CP05 peptide (CRHSQMTVTSRL) was synthesized and purchased from Ruixi biotechnology. 100 mg mPEG2K-TK-mal was weighed and dissolved in 5 mL DMF. Then CP05 peptide (1.1 eq) was added into the mixture and dissolved completely (react at room temperature for 12 h). Then the reaction solution was transferred into a dialysis bag (with a molecular weight cut-off of 3500 Da) and dialyzed in pure water for 24 h. The dialysis solution was collected and freeze-dried to obtain the product. ^1^H NMR spectra were recorded on a AVANCE III HD 400M spectrometer with DMSO as the solvent.

### Preparation of Stealth Exo

Exosomes (200 μg) derived from 293T cells were preincubated with mPEG2000-TK-CP05 (600 μg) overnight at 4 °C, followed by washing with PBS for three times and centrifugation to remove unbound mPEG2000-TK-CP05.

### miRNA loading into exosomes

Exosomes were electroporated with Cel-miR-54 (GenePharma, China) at 700 V, 150 μF in 4 mm electroporation cuvettes. The exosomes/miRNA ratio was about 10:1. Cel-miR-54 sequence is listed in Additional file 1: Table S2. After electroporation, exosomes were placed on the ice for 30 min to recover the membrane. Unloaded miRNAs attaching to the exosome surface were digested with RNase.

### RNA isolation and qRT-PCR

Total RNA of the sample was extracted using Trizol (Invitrogen, 15596018) as instructed. For miRNA, 2 μg of RNA was reverse transcribed into cDNA using miRcute Plus miRNA qPCR detection kit (Tiangen). For mRNA, 2 μg of RNA was reverse transcribed into cDNA by SMART® MMLV Reverse Transcriptase (Takara). qPCR was performed with FastStart Essential DNA Green Master Kit (06924204001, Roche). Relative expression of miRNA and mRNA was respectively normalized to U6 and GAPDH levels and calculated by 2^–ΔΔCt^. The PCR primer sequences are supplied in Additional file 1: Table S1.

### Exosome labeling and tracking in vitro

Exosomes (about 1 μg μL^−1^ at protein concentration) were incubated with 1 mM DiO (Beyotime) at the volume ratio of 500:1 for 20 min in 37 °C dark room. Free dyes were then removed by PBS washing and another round of exosome isolation. About 50 μg of dye-labeled exosomes were incubated with different groups of cells for 3 h. The cells were then washed with PBS three times and fixed with 4% PFA for 15 min and again washed with PBS for 3 times. The cell nuclei were stained with Hoechst33342 (1:1000, Beyotime) for 10 min at 37 °C. At the end of the experiment, the cells were washed with sodium acetate solution (to remove the nonspecific adhesion) and the images were captured by a Nikon A1 Spectral Confocal Microscope (Nikon, Japan).

### Exosome labeling and tracking in vivo

Exosomes (about 1 μg μL^−1^ at protein concentration) were incubated with 1 mM DiO (Beyotime), or DiR (Invitrogen) at the volume ratio of 500:1 for 20 min in 37 °C dark room. Free dyes were then removed by PBS washing and another round of exosome isolation. About 200 μg of dye-labeled exosomes was tail-vein injected into mice with indicated treatments. Exosome localization in the individual organs was detected by an IVIS Lumina II in vivo imaging system (PerkinElmer, Thermo Fisher, US) 4 h after injection. For test of the sliced section, mice were injected with DiO-labeled exosomes. The fresh organs were washed and embedded in optimal cutting temperature compound. Then, the embedded tissues were sliced into 8 μm sections. The tissue section was then stained with 1 μg mL^−1^ Hoechst 33342 for 10 min. Next, the tissue sections were observed and captured by Nikon A1 Spectral Confocal Microscope.

### Drugs loading into exosomes

Different concentration of Doxorubicin (Yuanye Biology, China) and Rose Bengal (Sigma-Aldrich, USA) were added to the exosomes (1 mg mL^−1^) and the mixture was sonicated (10% amplitude, 6 cycles, 8 s on/off, 2 min duration) by Ultrasonic Homogenizer (Scientz, China). The mixture was then incubated at 4 °C for 2 h to recover the exosome membrane to form drug loaded exosomes (Natural Exo@RB/Dox). Then Natural Exo@RB/Dox was obtained by centrifugation and free Dox and RB were discarded with the supernatant.

### Determination of encapsulation efficiency

Dox and RB were dissolved in PBS at a concentration of 10 mM and stored in − 20 °C at dark environment. The UV–vis absorption intensity of drugs was measured by the Nanodrop-2000 spectrophotometer (Thermo Scientific, USA) and the absorption intensity at the maximum absorption peak was used to calculate the drug concentration. The corresponding drug concentration standard curve was made according to the OD value of different concentrations of drugs at the maximum absorption peak. To measure the maximum drug loading dose in exosomes, different dose of drug was added to the exosomes (1 mg mL^−1^, 1 mL, protein level) (exosome group) and the same dose drug was added to PBS (free drug group). The OD value in free drug group was measured (A1). After sonication, the mixture was incubated at 4 °C and centrifuged, the absorption intensity of supernatant was measured (A2). Encapsulation efficiency was calculated by the following equation: Encapsulation rate = (A1 − A2)/A1 × 100%.

### In vitro cell experiments

For in vitro antitumor activities, A20 cells were seeded into 6-well plates at a density of 3 × 10^5^ cells per well (n = 3) and incubated with different samples (Stealth Exo, Stealth Exo@RB, Stealth Exo@RB/Dox) for 12 h. Then cells in laser group were followed exposure to a 532 nm laser (0.1 W cm^−2^, 5 min). 30 min later, the cells in different groups were collected for ROS level test, Flow cytometry and qPCR test.

### In vivo therapy

A20 tumor-bearing mice were randomly divided into 3 groups: PBS group, Stealth Exo@RB/Dox group, Stealth Exo@RB/Dox + laser group. When the tumors reached about 200 mm^3^, the mice were treated with PBS and Stealth Exo@RB/Dox via tail vein injection every 4 days for 4 times. A 532 nm laser irradiation (0.5 W cm^−2^, 10 min) was applied in the laser irradiation groups at 6 h, 12 h, 24 h post injection. Tumor size were monitored every 4 days. Mice were euthanized when major axis of tumor exceeded 20 mm or at the 60 day after tumor bearing. After treatment, mice were euthanized, the liver, heart, kidney and tumor were collected for H&E staining, TUNEL staining, C11 BODIPY 581/591 staining, DCFH staining. The rest of tumor tissues were collected for qPCR test.

### HE staining

The mice were euthanized and the thorax was opened and perfused with 4% paraformaldehyde from the apex of the mouse. After perfusion, the heart, liver, and kidney and tumor issues of mice were removed and soaked in 4% paraformaldehyde for 24 h. The tissues were placed in the embedding box and rinsed with running water for 30 min. After dehydration, transparency, waxing, embedding, sectioning, and spreading, staining was performed with hematoxylin and eosin (Beyotime, China).

### TUNEL staining

The tumor tissues and other organs were embedded with O.C.T and cut into 8 μm thick. All the experimental process was performed according to the instructions of the TUNEL staining kit (Beyotime, China). The nuclei were stained with 1 μg mL^−1^ Hoechst 33342. TUNEL positive cells of tumor tissue was observed by confocal microscope.

### ROS detection

A20 cells with density of 10^4^ cells mL^−1^ were seeded on glass bottom cell culture dish. Treated cells were incubated with 1 μM DCFH-DA (S0033, Beyotime) for 20 min at 37 °C, then washed with PBS three times and subsequently followed by nuclei staining with 1 μg mL^−1^ Hoechst 33342 for 10 min. Images were then captured by Nikon A1 Spectral Confocal Microscope (Nikon, Japan). In vivo ROS level test, the tumors tissues were stored in the optimal cutting temperature (O.C.T) specimen matrix (SAKURA, USA) for cryosections at − 20 °C by cryostat (Leica, USA). Frozen tumor tissue sections (about 8 μm thick) were stained with 1 μM DCFH-DA at 37 °C for 30 min, washed with PBS three times. Then tissue sections were stained with 1 μg mL^−1^ Hoechst 33342 for 10 min. The images were observed under Nikon A1 Spectral Confocal Microscope (Nikon, Japan).

### Ferroptosis detection

In order to test the lipid peroxidation level of tumor tissues, the tumors tissues were stored in the optimal cutting temperature (O.C.T) specimen matrix (SAKURA, USA) for cryosections at − 20 °C by cryostat (Leica, USA). Frozen tumor tissue sections (about 8 μm thick) were stained with 2 μM C11 BODIPY 581/591 at 37 °C for 30 min, washed with PBS three times. Then tissue sections were stained with 1 μg mL^−1^ Hoechst 33342 for 10 min. The images were observed under 488 nm excitation of Nikon A1 Spectral Confocal Microscope (Nikon, Japan).

### Statistical analysis

Data and results in this study are expressed as mean ± SEM as indicated. Student’s t-test was used for two group comparison while one-way ANOVA test was used to compare the differences between more than three groups. Significance for animal survival was analyzed by Kaplan–Meier with log-rank analysis. P values of < 0.05 indicate statistical difference.

## Supplementary Information

**Additional file 1: Figure S1.** ^1^H NMR spectra of CP05 and mPEG2K-TK-mal. (A) ^1^H NMR spectra (DMSO as solvent) of CP05. (B) ^1^H NMR spectra (DMSO as solvent) of mPEG2K-TK-mal. (C) Structural formula of CP05-TK-mPEG2000. **Figure S2.** Ex vivo imaging analysis of the MPS escape efficiency of Stealth Exo. (A) Representative in vitro fluorescent images show DiR-labeled exosomes uptake in main organs at 4 h/12 h/24 h after injection. n = 6 mice in each group. (B) Quantification of fluorescence intensity of different organs. Data are presented as the mean ± SEM, n = 5. **Figure S3.** Fluorescence microscopy analysis of the MPS escape efficiency of Stealth Exo. Dio-labeled exosomes (green) were visualized by fluorescence microscopy. Nuclei were counterstained with Hoechst. Scale bar = 200 μm. **Figure S4.** Efficient loading of RB into exosomes. (A) Schematic illustration of the loading procedure of RB. (B) UV–vis absorption spectra of RB, maximum at 540 nm. (C) Absorbance vs. Concentration calibration curve for RB, with the regression coefficient of 0.9987 for RB. (D) Loading efficiency curve showing the various loading rate of RB at different concentrations. Exos were incubated with different doses of RB (500 μg mL^−1^ exosomes at protein level). **Figure S5.** Effects of laser irradiation on the morphology and size of Stealth Exo. (A) Representative images of transmission electron microscopy of Stealth Exo@RB irradiated by 532 nm laser (0.1 W cm^−2^, 5 min). (B) Particle size distribution of Stealth Exo after 532 nm laser irradiation as analyzed by DLS (0.1 W cm^−2^, 5 min). **Figure S6.** Local laser irradiation induces burst release of Stealth Exo in the tumor tissue. (A) Dio-labeled exosomes (green) were visualized by fluorescence microscopy. Nuclei were counterstained with Hoechst. Scale bar = 200 μm. (B) Semi-quantification of fluorescence intensity of different organs. Data are presented as the mean ± SEM, n = 5. **Figure S7.** Efficient loading of Dox into exosomes. (A) Schematic illustration of the loading procedure of Dox. (B) UV–vis absorption spectra of Dox, maximum at 480 nm. (C) Absorbance vs. Concentration calibration curve for Dox, with the regression coefficient of 0.9841 for Dox. (D) Loading efficiency curve showing the various loading rate of Dox at different concentrations. Exos were incubated with different doses of RB (500 μg mL^−1^ exosomes at protein level). **Figure S8.** Effects of 532 nm laser irradiation on apoptosis and ferroptosis of A20 cells. (A) ROS detection by DCFH-DA staining in A20 cells treated with or without laser irradiation. scale bar = 100 μm. (B) FCM analysis of cell death in A20 cells treated with or without laser irradiation. (C) qPCR analysis of ferroptosis related genes in A20 cells treated as above. *Gapdh* served as internal controls. Data are expressed as mean ± SEM of three different experiments. **Figure S9.** Stealth Exo@RB/Dox induces ferroptosis in tumor tissues upon laser irradiation. Representative C11 BODIPY 581/591 staining images of the tumor tissues from A20-bearing mice with indicated treatment groups. Scale bar = 200 μm, n = 6 for each group. **Figure S10.** Immune activation upon Stealth Exo@RB/Dox treatment. Expression level of T cell activation cytokines *Ifn-γ* (A), *Il-2* (B), *Tnf-α* (C), *Il-6* (D) and T cell suppression cytokines *Tgf-β* (E), *Il-10* (F) in tumor issues of A20-bearing mice with indicated treatments. Data shown are representative of 6 mice. *p < 0.05. **Figure S11.** The stability of the stealth exosome and cell viability after treatment. (A) Zeta potentials of Stealth Exo stored in 4 °C for 0 day or 10 days. (B) Particle size distribution of Stealth Exo stored in 4 °C for 0 day or 10 days as measured by DLS. (C) Plasma Epi-fluorescence measured at different time points after DiR labelled exosomes injection. (D) Cell viability measured by CCK8 assay after different treatments. **Table S1.** Primers used in the study. **Table S2.** siRNAs/miRNAs used in the study.

## Data Availability

All the data used/analyzed in the current study are available from the corresponding author on reasonable request.

## Abbreviations

ROS: Reactive oxygen species
RB: Rose Bengal
Dox: Doxorubicin
Exo: Exosome
PEG: Polyethylene glycol
DLBCL: Diffuse large B-cell lymphoma
NHL: Non-Hodgkin lymphoma
MPS: Mononuclear phagocyte system
EPR: Enhanced permeability and retention
NP: Nanoparticles
TK: Thioketal
PDT: Photodynamic therapy
PS: Photosensitizers
