# Supplementary material for: ROS triggered local delivery of stealth exosomes to tumors for enhanced chemo/photodynamic therapy
Source: J Nanobiotechnology. 2022 Aug 23;20:385. doi: 10.1186/s12951-022-01591-7 (PMC9400243; doi:10.1186/s12951-022-01591-7)
Supplement: Supplementary file 1 — Additional file 1: Figure S1. 1H NMR spectra of CP05 and mPEG2K-TK-mal. (A) 1H NMR spectra (DMSO as solvent) of CP05. (B) 1H NMR spectra (DMSO as solvent) of mPEG2K-TK-mal. (C) Structural formula of CP05-TK-mPEG2000. Figure S2. Ex vivo imaging analysis of the MPS escape efficiency of Stealth Exo. (A) Representative in vitro fluorescent images show DiR-labeled exosomes uptake in main organs at 4 h/12 h/24 h after injection. n = 6 mice in each group. (B) Quantification of fluorescence intensity of different organs. Data are presented as the mean ± SEM, n = 5. Figure S3. Fluorescence microscopy analysis of the MPS escape efficiency of Stealth Exo. Dio-labeled exosomes (green) were visualized by fluorescence microscopy. Nuclei were counterstained with Hoechst. Scale bar = 200 μm. Figure S4. Efficient loading of RB into exosomes. (A) Schematic illustration of the loading procedure of RB. (B) UV–vis absorption spectra of RB, maximum at 540 nm. (C) Absorbance vs. Concentration calibration curve for RB, with the regression coefficient of 0.9987 for RB. (D) Loading efficiency curve showing the various loading rate of RB at different concentrations. Exos were incubated with different doses of RB (500 μg mL−1 exosomes at protein level). Figure S5. Effects of laser irradiation on the morphology and size of Stealth Exo. (A) Representative images of transmission electron microscopy of Stealth Exo@RB irradiated by 532 nm laser (0.1 W cm−2, 5 min). (B) Particle size distribution of Stealth Exo after 532 nm laser irradiation as analyzed by DLS (0.1 W cm−2, 5 min). Figure S6. Local laser irradiation induces burst release of Stealth Exo in the tumor tissue. (A) Dio-labeled exosomes (green) were visualized by fluorescence microscopy. Nuclei were counterstained with Hoechst. Scale bar = 200 μm. (B) Semi-quantification of fluorescence intensity of different organs. Data are presented as the mean ± SEM, n = 5. Figure S7. Efficient loading of Dox into exosomes. (A) Schematic illustration o [file 12951_2022_1591_MOESM1_ESM.docx]

Supporting Information

**ROS triggered local delivery of stealth exosomes to tumors for enhanced chemo/photodynamic therapy**

Zhuo Wan^1,5,6 #^, Xueqi Gan^2, #^, Ruiyan Mei^1,5,6 #^, Jianbin Du^3^, Wen Fan^1,5,6^, Mengying Wei^4^, Guodong Yang^4^, Weiwei Qin^1,5,6 *^, Zhuoli Zhu^2, *^, Li Liu^1,5,6 *^


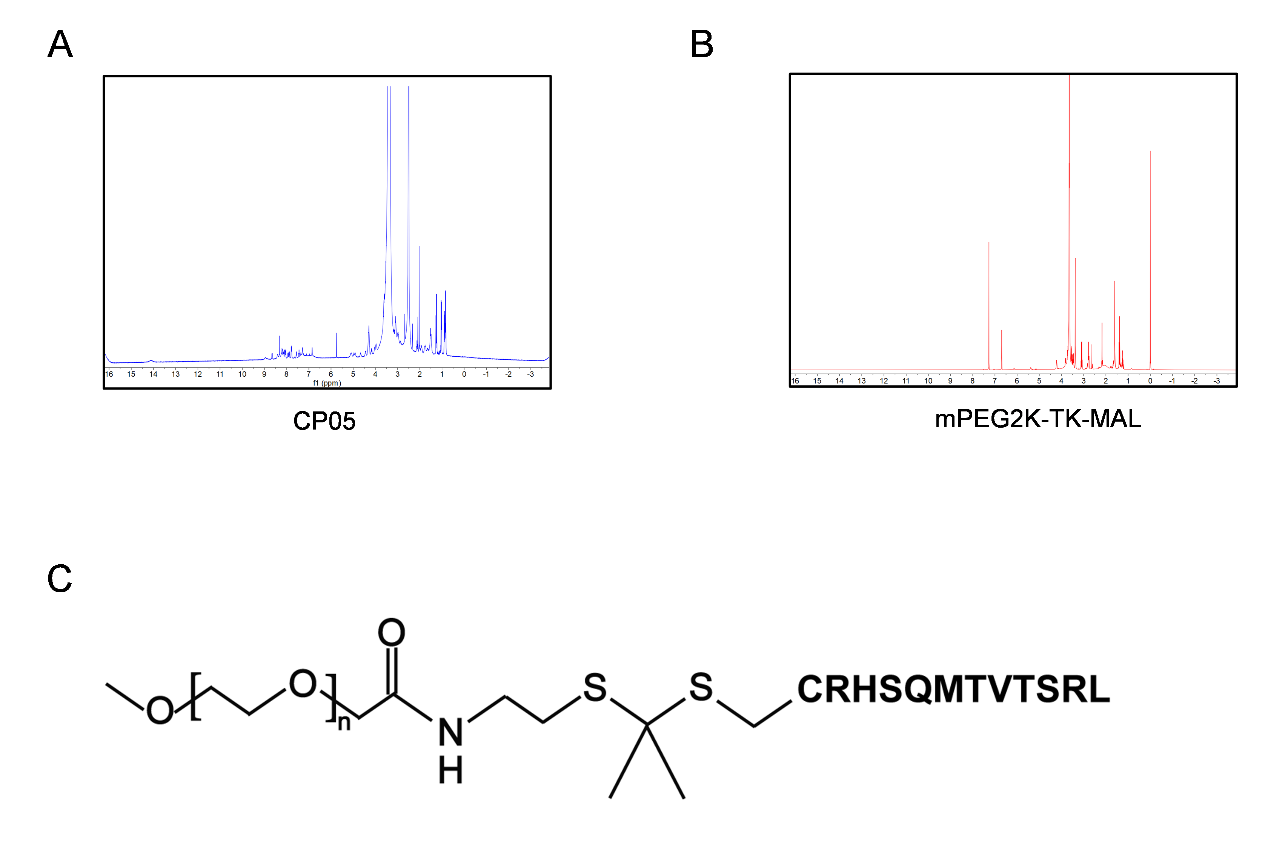


**Figure S1.** ^1^H NMR spectra of CP05 and mPEG2K-TK-mal. (A) ^1^H NMR spectra ((DMSO as solvent) of CP05. (B) ^1^H NMR spectra ((DMSO as solvent) of mPEG2K-TK-mal. (C) Structural formula of CP05-TK-mPEG2000.


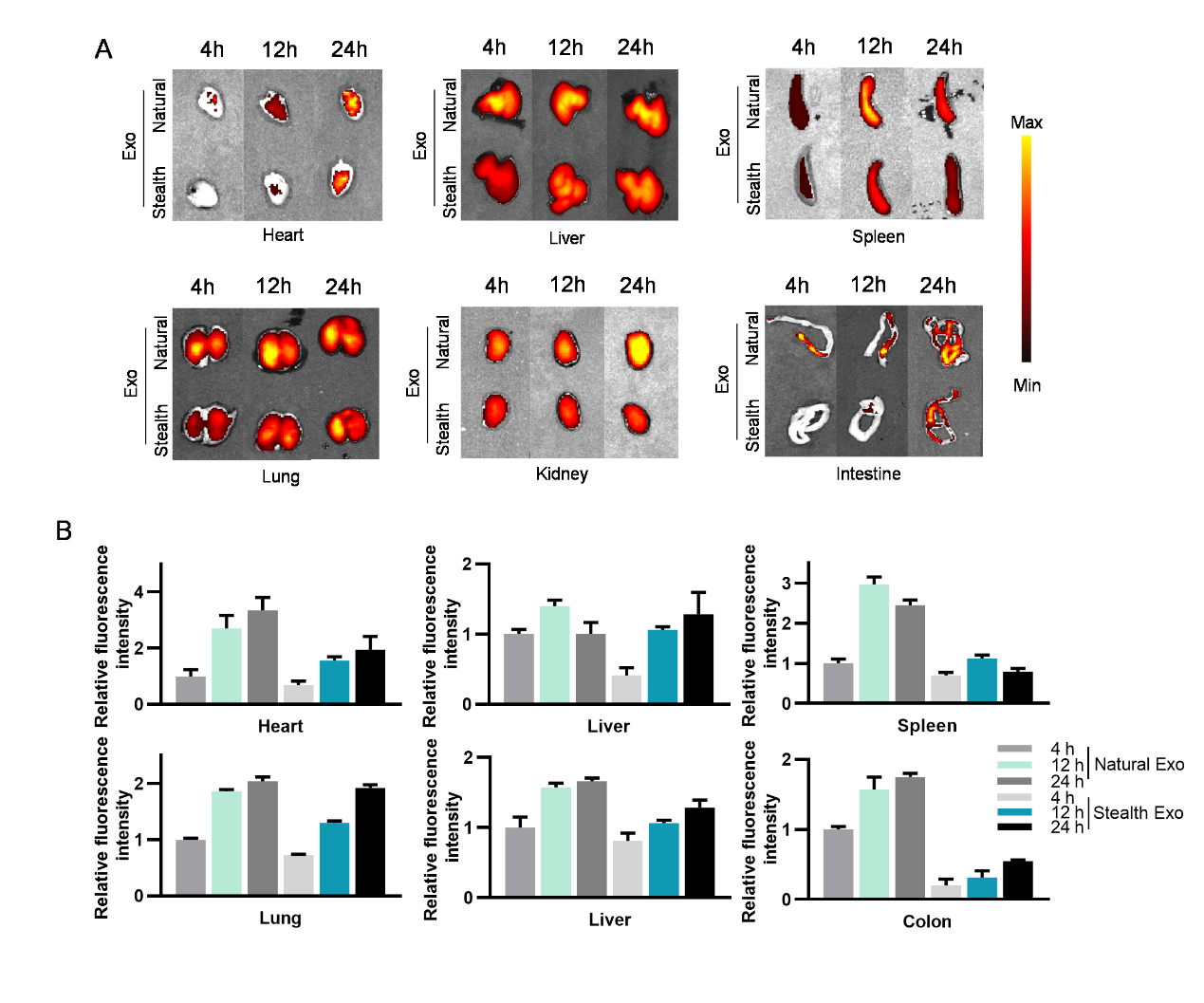


**Figure S2.** Ex vivo imaging analysis of the MPS escape efficiency of Stealth Exo. (A) Representative in vitro fluorescent images show DiR-labeled exosomes uptake in main organs at 4 h/12 h/24 h after injection. n=6 mice in each group. (B) Quantification of fluorescence intensity of different organs. Data are presented as the mean ± SEM, n=5.


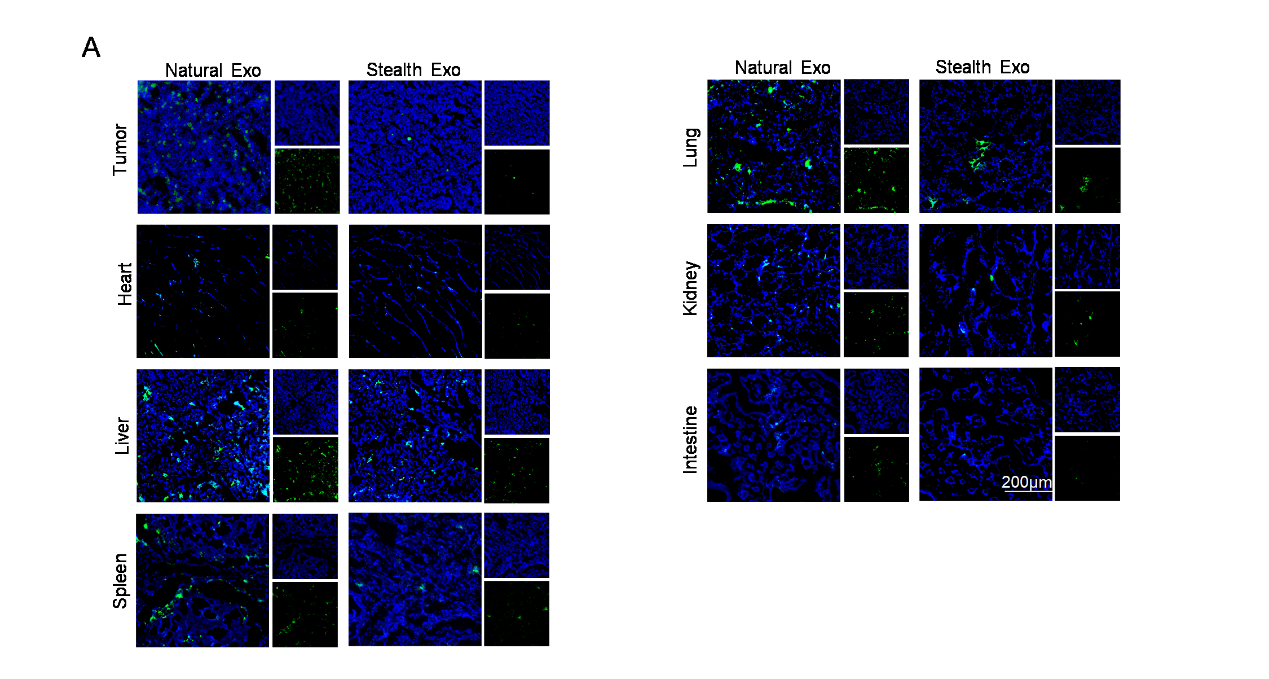


**Figure S3.** Fluorescence microscopy analysis of the MPS escape efficiency of Stealth Exo. Dio-labeled exosomes (green) were visualized by fluorescence microscopy. Nuclei were counterstained with Hoechst. Scale bar=200 μm.


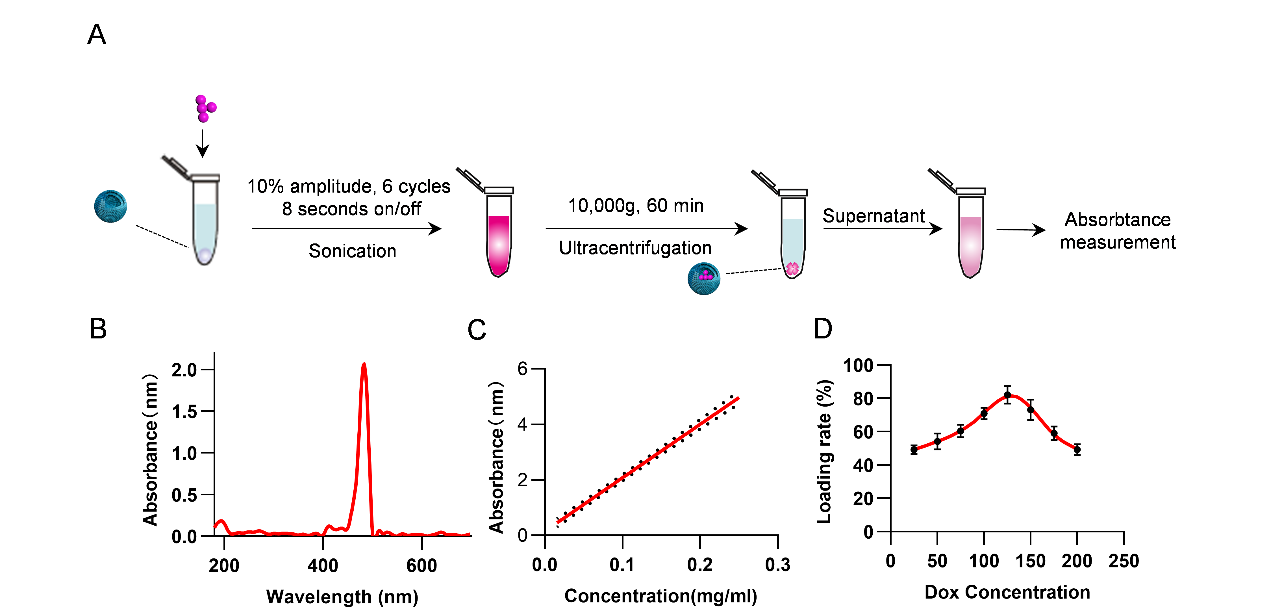


**Figure S4.** Efficient loading of RB into exosomes. (A) Schematic illustration of the loading procedure of RB. (B) UV-vis absorption spectra of RB, maximum at 540 nm. (C) Absorbance vs. Concentration calibration curve for RB, with the regression coefficient of 0.9987 for RB. (D) Loading efficiency curve showing the various loading rate of RB at different concentrations. Exos were incubated with different doses of RB (500 μg ml^-1^ exosomes at protein level).


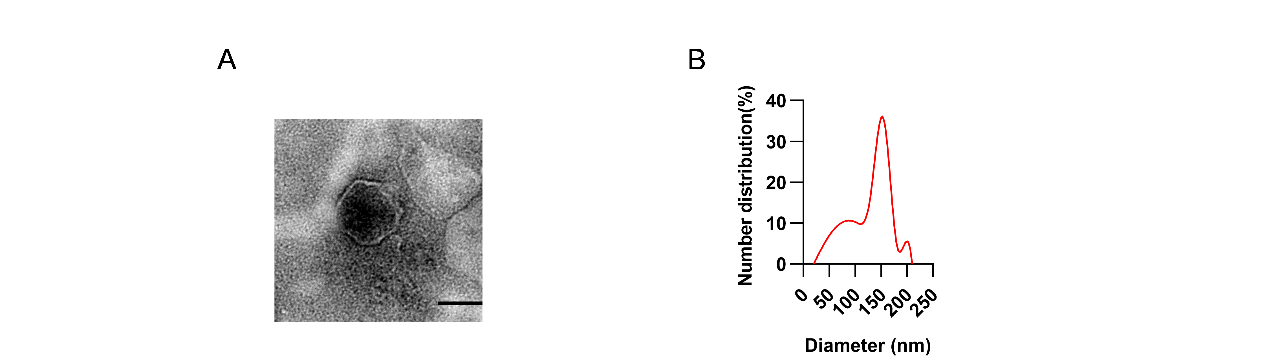


**Figure S5.** Effects of laser irradiation on the morphology and size of Stealth Exo. (A) Representative images of transmission electron microscopy of Stealth Exo@RB irradiated by 532 nm laser (0.1W cm^-2^, 5 min). (B) Particle size distribution of Stealth Exo after 532 nm laser irradiation as analyzed by DLS (0.1W cm^-2^, 5 min).


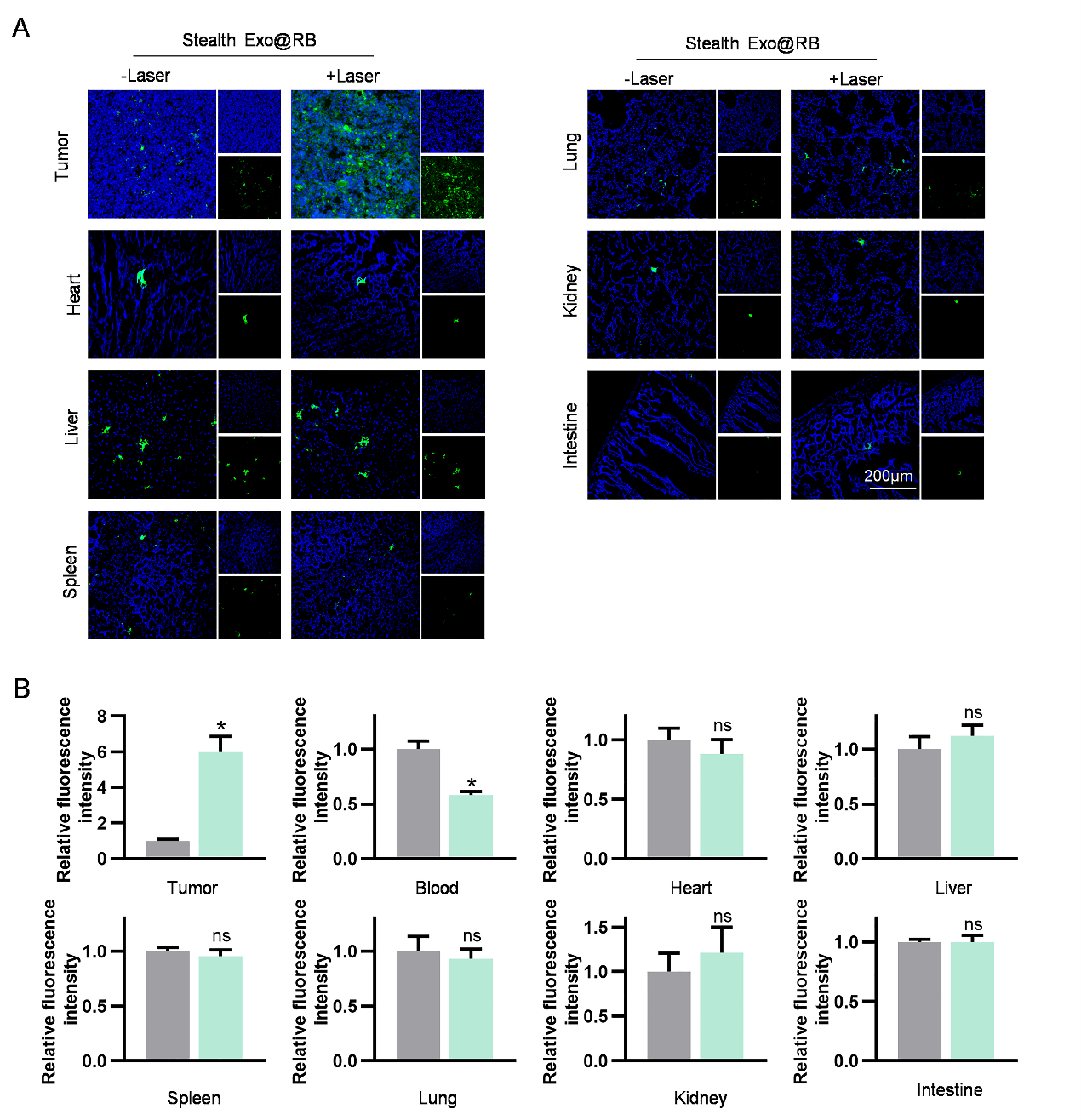


**Figure S6.** Local laser irradiation induces burst release of Stealth Exo in the tumor tissue. (A) Dio-labeled exosomes (green) were visualized by fluorescence microscopy. Nuclei were counterstained with Hoechst. Scale bar=200 μm. (B) Semi-quantification of fluorescence intensity of different organs. Data are presented as the mean ± SEM, n=5.


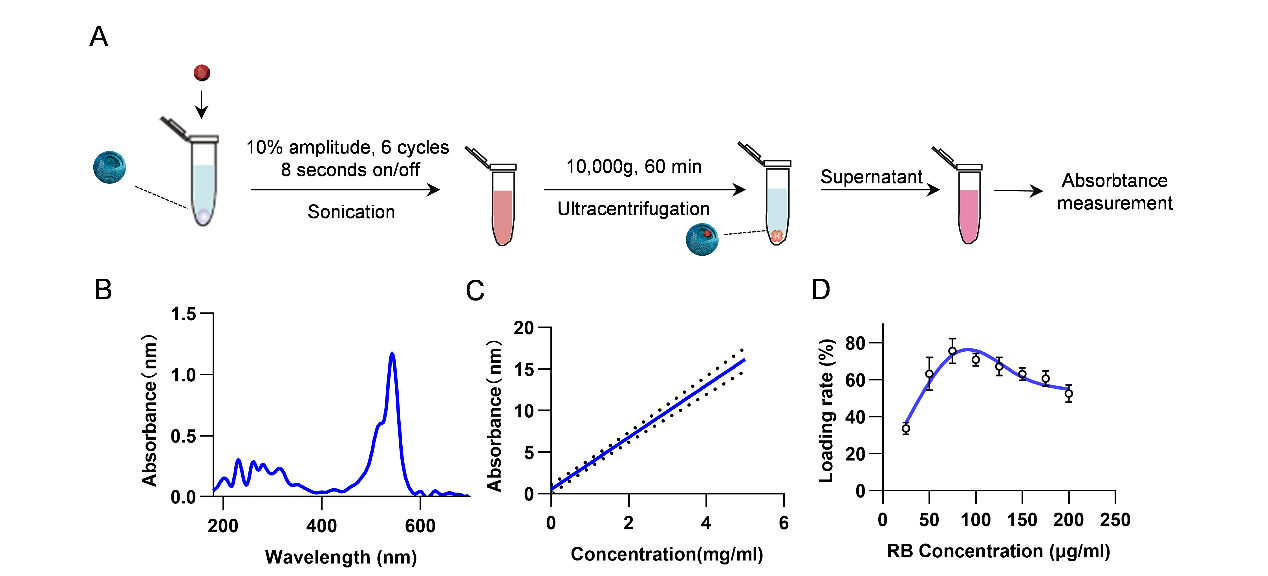


**Figure S7.** Efficient loading of Dox into exosomes. (A) Schematic illustration of the loading procedure of Dox. (B) UV-vis absorption spectra of Dox, maximum at 480 nm. (C) Absorbance vs. Concentration calibration curve for Dox, with the regression coefficient of 0.9841 for Dox. (D) Loading efficiency curve showing the various loading rate of Dox at different concentrations. Exos were incubated with different doses of RB (500 μg ml^-1^ exosomes at protein level).


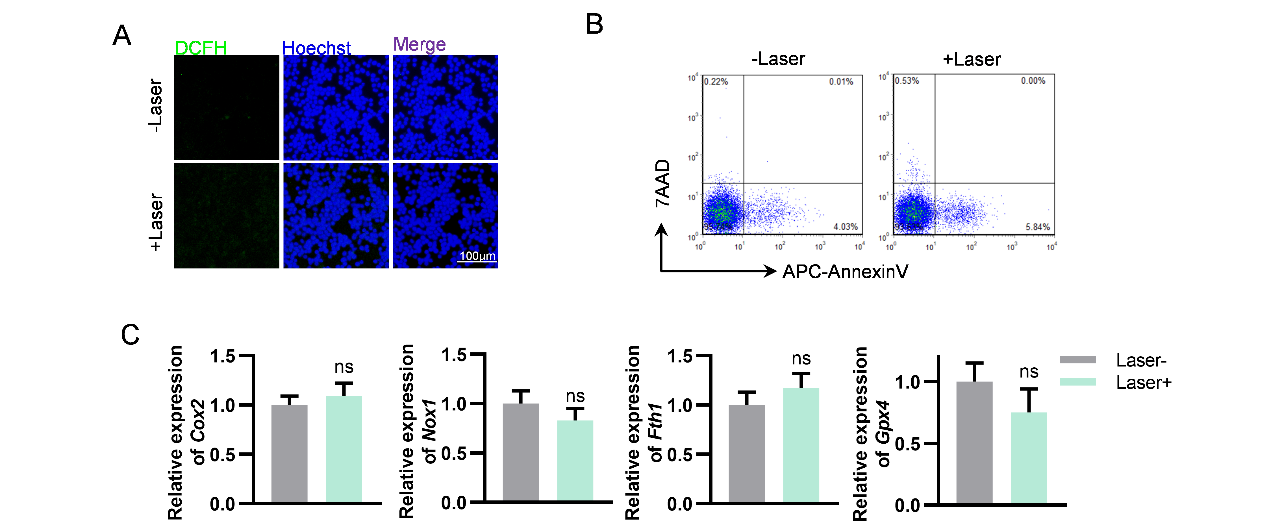


**Figure S8.** Effects of 532 nm laser irradiation on apoptosis and ferroptosis of A20 cells. (A) ROS detection by DCFH-DA staining in A20 cells treated with or without laser irradiation. scale bar=100 μm. (B) FCM analysis of cell death in A20 cells treated with or without laser irradiation. (C) qPCR analysis of ferroptosis related genes in A20 cells treated as above. *Gapdh* served as internal controls. Data are expressed as mean ± SEM of three different experiments.


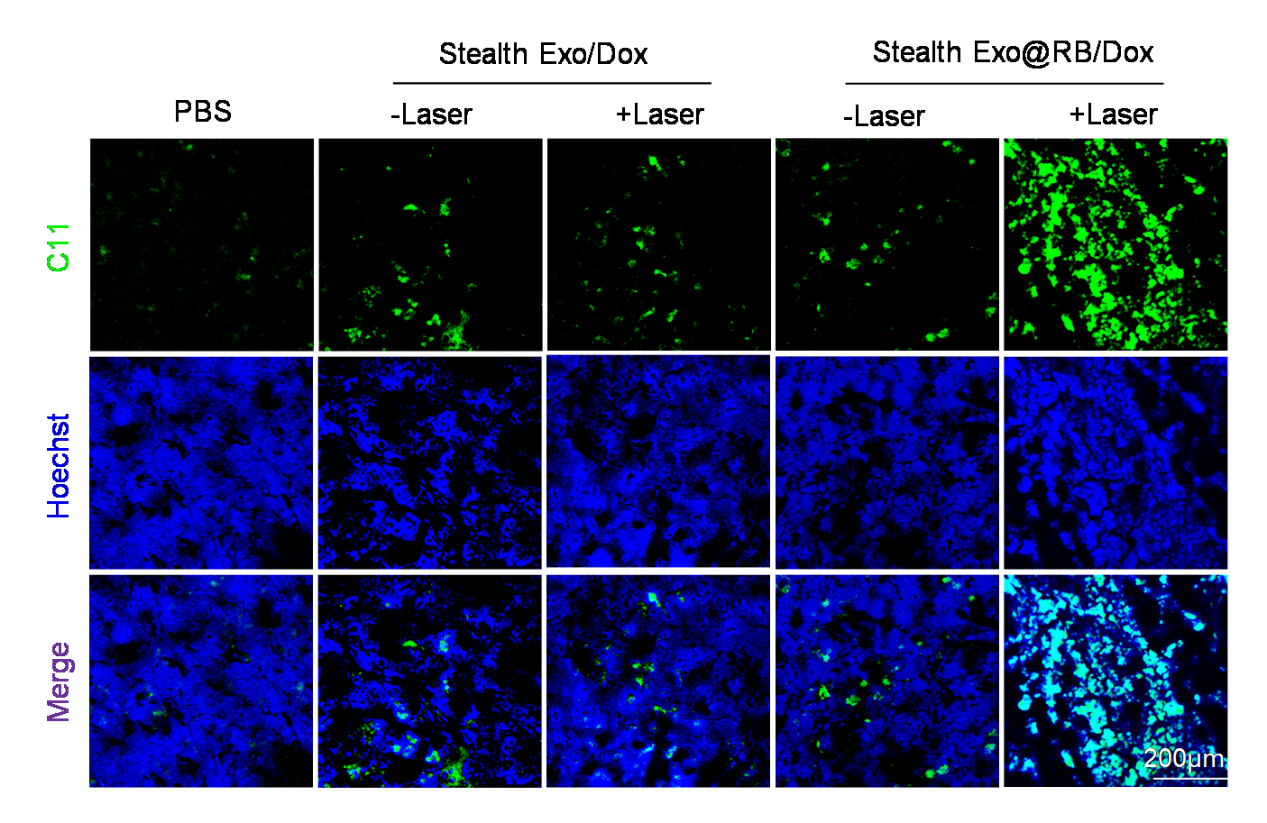


**Figure S9.** Stealth Exo@RB/Dox induces ferroptosis in tumor tissues upon laser irradiation. Representative C11 BODIPY 581/591 staining images of the tumor tissues from A20-bearing mice with indicated treatment groups. Scale bar=200 μm, n=6 for each group.


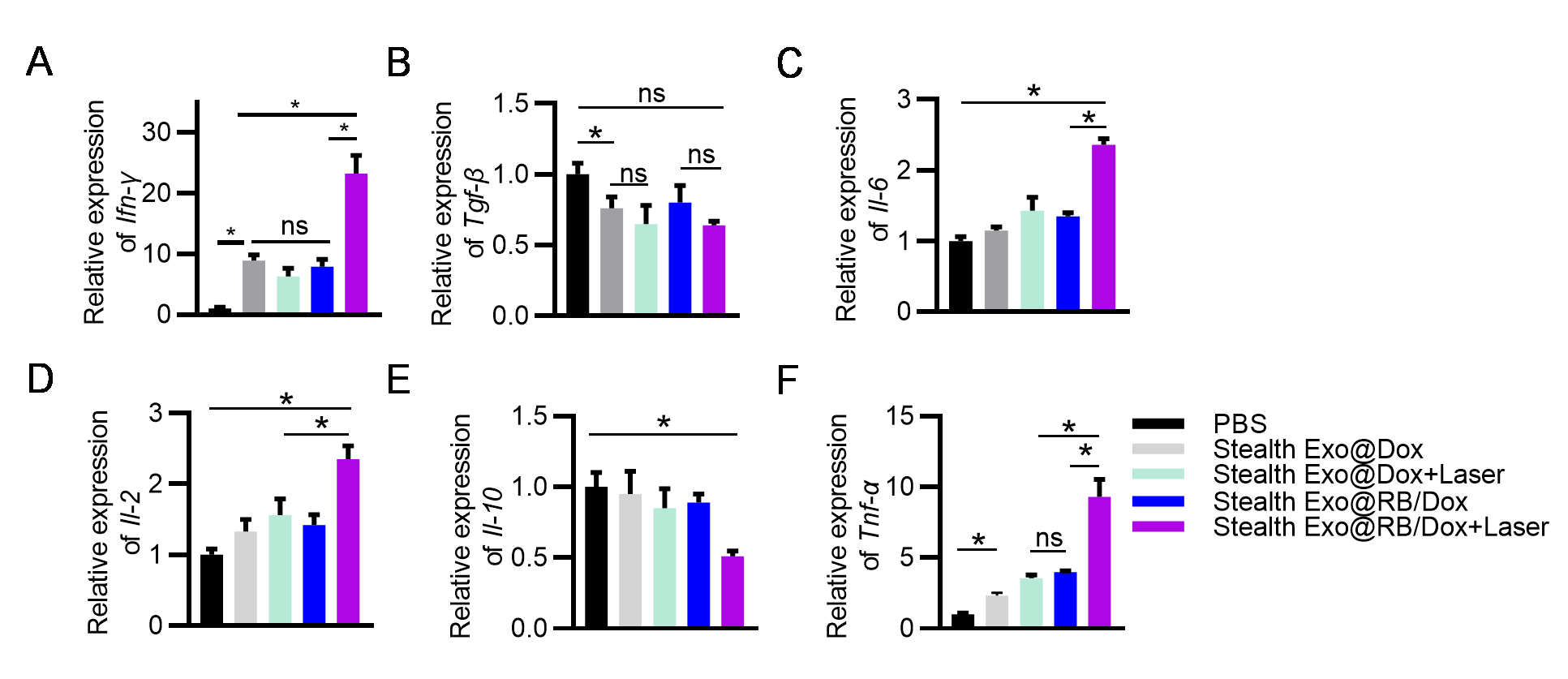


**Figure S10.** Immune activation upon Stealth Exo@RB/Dox treatment. Expression level of T cell activation cytokines *Ifn-γ* (A), *Il-2* (B), *Tnf-α* (C), *Il-6* (D) and T cell suppression cytokines *Tgf-β* (E), *Il-10* (F) in tumor issues of A20-bearing mice with indicated treatments. Data shown are representative of 6 mice. *, p < 0.05.


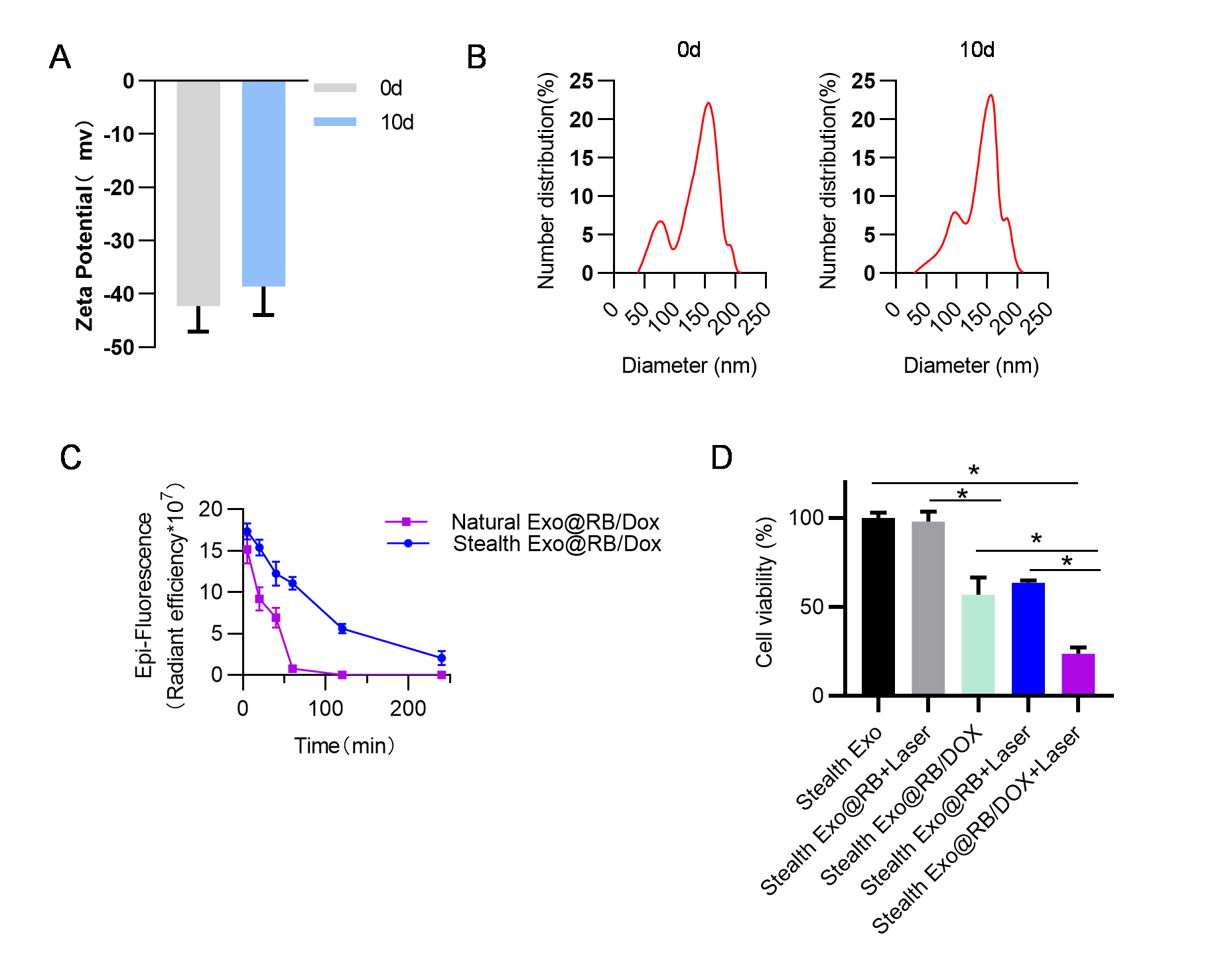


**Figure S11.** The stability of the stealth exosome and cell viability after treatment. (A) Zeta potentials of Stealth Exo stored in 4$℃$ for 0 day or 10 days. (B) Particle size distribution of Stealth Exo stored in 4$℃$ for 0 day or 10 days as measured by DLS. (C) Plasma Epi-fluorescence measured at different time points after DiR labelled exosomes injection. (D) Cell viability measured by CCK8 assay after different treatments.

**Table S1. Primers used in the study.**

| Gene | Forward primer | Reverse primer |
| --- | --- | --- |
| *Il-2* | TGAGCAGGATGGAGAATTACAGG | GTCCAAGTTCATCTTCTAGGCAC |
| *Il-6* | TAGTCCTTCCTACCCCAATTTCC | TTGGTCCTTAGCCACTCCTTC |
| *Il-10* | GCTCTTACTGACTGGCATGAG | CGCAGCTCTAGGAGCATGTG |
| *Tnf-α* | CCGGGAGAAGAGGGATAGCTT | TCGGACAGTCACTCACCAAGT |
| *Tgf-β* | CTCCCGTGGCTTCTAGTGC | GCCTTAGTTTGGACAGGATCTG |
| *Inf-γ* | ATGAACGCTACACACTGCATC | CCATCCTTTTGCCAGTTCCTC |
| *Gpx4* | CGCAGCTCTAGGAGCATGTG | CCCTGTACTTATCCAGGCAGA |
| *Fth1* | CAAGTGCGCCAGAACTACCA | ACAGATAGACGTAGGAGGCATAC |
| *Cox2* | AACCCAGGGGATCGAGTGT | CGCAGCTCAGTGTTTGGGAT |
| *Nox1* | GCTGGATTTGAGAGCGTTGC | GGTGGTATCTAGGGCTATGCT |
| *Gapdh* | AGGTCGGTGTGAACGGATTTG | GGGGTCGTTGATGGCAACA |
| *U6* | GGATGACACGCAAATTCGTGAA | Provided in the kit |
| *Cel-miR-54* | AGGATATGAGACGACGAGAACA | Provided in the kit |

**Table S2. siRNAs/miRNAs used in the study.**

| Name | sense | antisense |
| --- | --- | --- |
| *Cel-miR-54* | AGGAUAUGAGACGACGAGAACA | UUCUCGUCGUCUCAUAUCCUUU |
